# Non-random Mis-segregation of Human Chromosomes

**DOI:** 10.1016/j.celrep.2018.05.047

**Published:** 2018-06-13

**Authors:** Joseph Thomas Worrall, Naoka Tamura, Alice Mazzagatti, Nadeem Shaikh, Tineke van Lingen, Bjorn Bakker, Diana Carolina Johanna Spierings, Elina Vladimirou, Floris Foijer, Sarah Elizabeth McClelland

**Affiliations:** 1 Barts Cancer Institute, Queen Mary University of London, London EC1M 6BQ, UK; 2 European Research Institute for the Biology of Ageing, University of Groningen, University Medical Center Groningen, A. Deusinglaan 1, Groningen 9713, the Netherlands; 3 UCL Cancer Institute, University College London, 72 Huntley Street, London WC1E 6DD, UK

**Keywords:** ImageStream, chromosome mis-segregation, cohesion fatigue, aneuploidy

## Abstract

A common assumption is that human chromosomes carry equal chances of mis-segregation during compromised cell division. Human chromosomes vary in multiple parameters that might generate bias, but technological limitations have precluded a comprehensive analysis of chromosome-specific aneuploidy. Here, by imaging specific centromeres coupled with high-throughput single-cell analysis as well as single-cell sequencing, we show that aneuploidy occurs non-randomly following common treatments to elevate chromosome mis-segregation. Temporary spindle disruption leads to elevated mis-segregation and aneuploidy of a subset of chromosomes, particularly affecting chromosomes 1 and 2. Unexpectedly, we find that a period of mitotic delay weakens centromeric cohesion and promotes chromosome mis-segregation and that chromosomes 1 and 2 are particularly prone to suffer cohesion fatigue. Our findings demonstrate that inherent properties of individual chromosomes can bias chromosome mis-segregation and aneuploidy rates, with implications for studies on aneuploidy in human disease.

## Highlights

• Aneuploidy rates vary between chromosomes after drug-induced mis-segregation
• Chromosomes 1 and 2 comprise a large proportion of anaphase lagging chromosomes
• Mitotic delay and cohesion fatigue drive chromosome mis-segregation
• Chromosomes 1 and 2 are particularly prone to cohesion fatigue

## Graphical Abstract

**Figure:**
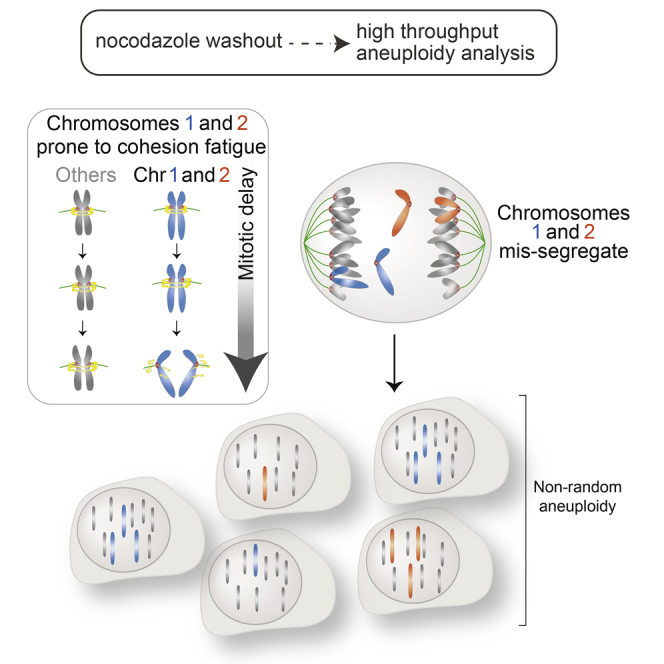

## Teaser

Worrall et al. show that individual human chromosomes can respond differently to defects in mitosis that lead to chromosome mis-segregation. Following nocodazole washout, chromosomes 1 and 2 are particularly prone to a weakening of centromeric cohesion and elevated rates of chromosome lagging during anaphase.

## Introduction

**Figure 1:**
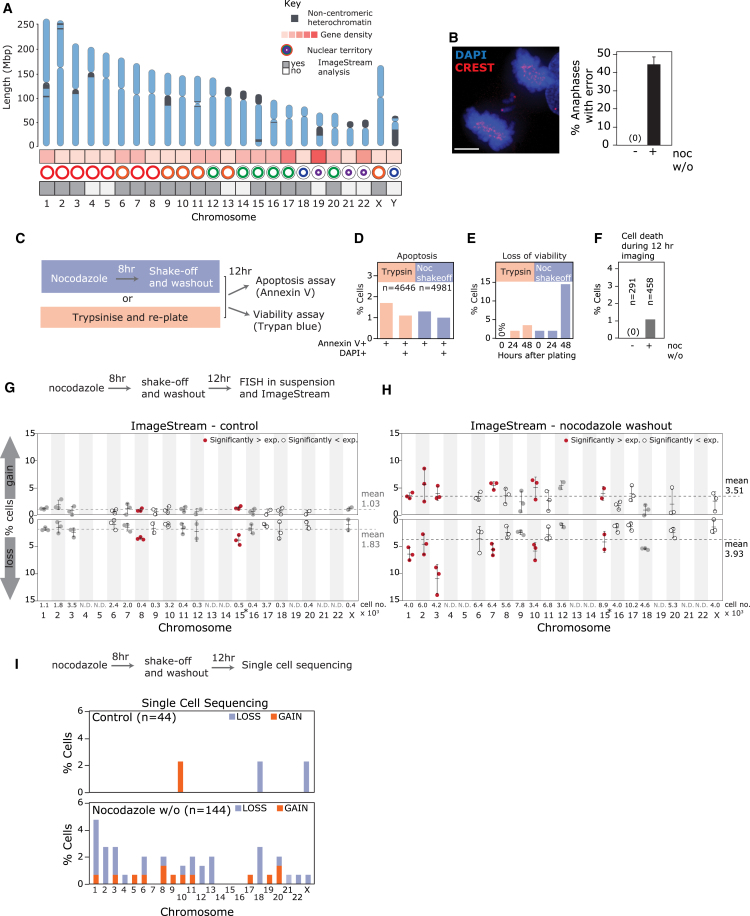
Chromosome Mis-segregation Induced by Nocodazole Washout Leads to Non-random Aneuploidy (A) Cartoon illustrating a selection of known chromosomal attributes (Cremer and Cremer, 2010). Gene density (number of genes divided by length of chromosome [Mb]) was divided equally into five groups. (B) Immunofluorescence image and quantification of segregation errors from RPE1 anaphase cells following nocodazole washout. Centromeres marked by CREST anti-sera. Mean and SD from three independent experiments is shown. Scale bar in this and all following images represents 5 μm. (C) Experimental workflow for (D)–(F). (D) Quantification of percentage annexin V^+^ (early apoptotic) and annexin V^+^ DAPI^+^ cells (late apoptotic) analyzed by flow cytometry. (E) Representative trypan blue cell viability assay of RPE1 cells treated with 8 hr nocodazole, then released for times indicated. (F) RPE1 cells stably expressing H2B-RFP were filmed following release from 8 hr nocodazole. Cell death rates were quantified from two independent movies. (G and H) ImageStream analysis of RPE1 cells untreated (G) or treated with nocodazole washout (H). Dots represent independent experiments. Red dots and open circles mark chromosomes with aneuploidy rates significantly higher and lower than expected, respectively, using chi-square analysis. Dashed lines indicate mean aneuploidy rates. Number of cells analyzed (×10^3^) per chromosome is indicated in lower box. Chromosome 15 is marked by an asterisk because it was identified as significantly more aneuploid than expected by chance in both conditions. Therefore we cannot exclude possible low-level stable aneuploidy for this chromosome. (I) Percentage cells exhibiting whole aneuploidy events were collated from SCS data analyzed using AneuFinder (four independent experiments; 44 control and 144 nocodazole washout treated cells in total). See also Figures S1–S3.

Aneuploidy—deviation from a multiple of the haploid chromosome number—is the leading cause of spontaneous miscarriage and birth defects in humans (Nagaoka et al., 2012) and represents a key hallmark of cancer, in which recurrent patterns of aneuploidy are observed (Ben-David et al., 2016, Duijf et al., 2013, Taylor et al., 2018). Human chromosomes vary widely in size, gene density, interphase nuclear territory, and heterochromatin distribution (Figure 1A; Table S1). However, the question of whether these or additional characteristics generate bias in mis-segregation rates has not been answered to date, because high-throughput methods to analyze chromosome-specific aneuploidy were lacking. The standard approach to measure aneuploidy, manual scoring of chromosome number using fluorescence *in situ* hybridization (FISH) of centromere-targeted probes is low throughput and subject to significant artifacts (Faggioli et al., 2012, Fenech, 2007, Knouse et al., 2014, Valind et al., 2013, van den Bos et al., 2016), limiting the resolution of previous efforts to examine biased mis-segregation (Brown et al., 1983, Evans and Wise, 2011, Fauth et al., 1998, Hovhannisyan et al., 2016, Spence et al., 2006, Torosantucci et al., 2009, Xi et al., 1997). New technologies such as next-generation sequencing-based methods (Bakker et al., 2016, van den Bos et al., 2016) are still expensive and technically challenging (Bakker et al., 2015, Gao et al., 2016, Knouse et al., 2014). To resolve this we analyzed individual chromosome aneuploidy rates in a high-throughput manner and in the absence of fitness effects and selection. We used the ImageStream^X^ cytometer to quantify FISH-marked centromeres in thousands of single cells, following induction of chromosome mis-segregation using nocodazole washout. We show that resulting aneuploidy in daughter cells is non-random and validate our findings using single-cell sequencing. Interestingly, chromosomes 1 and 2 are highly prone to lagging at anaphase following nocodazole washout, and this occurs in multiple non-transformed cell types. We find that these chromosomes are inherently susceptible to cohesion fatigue that results in elevated lagging at anaphase and aneuploidy in daughter cells.

## Results

### High-Throughput Screening Using the ImageStream^X^ Cytometer Reveals Non-random Aneuploidy following Induction of Chromosome Mis-segregation

We examined aneuploidy rates in diploid h-TERT-immortalized human retinal pigment epithelium cells (RPE1). Non-transformed human cells exhibit very low rates of spontaneous chromosome segregation errors, so we disrupted the fidelity of cell division to elevate chromosome mis-segregation and allow the detection of bias between chromosomes. We used a nocodazole shake-off and washout treatment to promote chromosome segregation errors (Figure 1B) due to formation of merotelic attachments (Cimini et al., 2001, Zhang et al., 2015), a key proposed driver of chromosome mis-segregation and aneuploidy in cancer (Bakhoum et al., 2009, Ertych et al., 2014). To determine aneuploidy rates independently of selection effects, we analyzed cells 12 hr after nocodazole washout and shake-off, verifying that this procedure does not affect cell viability (Figures 1C–1F, S1A, and S1B). Live-cell imaging and fluorescence-activated cell sorting (FACS)-based cell cycle profiling revealed that at this time point, cells have exited mitosis and are mainly in G1, without cell death or further division events that could influence population aneuploidy rates (Figures S1C–S1F; Video S1). We used the ImageStream^X^ Mark II cytometer (hereafter ImageStream), an imaging flow cytometer previously used to detect monosomy and trisomy in peripheral blood mononuclear cells with high accuracy (Minderman et al., 2012), to analyze aneuploidy frequencies of individual chromosomes marked with centromere-specific FISH probes. This approach has advantages over conventional FISH-based methods; a “FISH-in suspension” procedure improves signal-to-noise ratio, thousands of cells per sample are analyzed, and centromere number is determined using both automated spot counting and fluorescence intensity measurements (Minderman et al., 2012) (see Experimental Procedures; Figure S2). We were able to analyze the majority of the 23 human chromosomes except for a subset of human chromosomes that lacks sufficiently unique pericentromeric sequence to generate specific centromeric FISH probes (chromosomes 4, 5, 13, 14, 19, 21, and 22; Table S2). As expected we observed an increase in overall aneuploidy following nocodazole washout (Figures 1G and 1H). Chi-square testing revealed that aneuploidy rates varied more than expected if chromosome mis-segregation rates were equal between chromosomes (p < 10^−6^; see Supplemental Experimental Procedures). To identify specific chromosomes that deviated significantly from expected rates, we used post hoc binomial tests, Bonferroni corrected for multiple testing, which indicated that chromosomes 1, 2, 3, 7, and 10 were affected significantly more than expected following nocodazole washout (Figure 1H, red dots). A subset of chromosomes was also affected significantly less than expected (chromosomes 8, 11, 16, and X; Figure 1H, open circles). To validate ImageStream aneuploidy analysis, we performed single-cell sequencing (SCS) and aneuploidy detection using AneuFinder (Bakker et al., 2016), which corroborated elevated aneuploidy for chromosomes 1, 2, and 3 following nocodazole washout (Figures 1I and S3A). SCS did not detect elevated aneuploidy for chromosomes 7 or 10, potentially because of the smaller number of cells analyzed or an artifact of the ImageStream analysis. We noticed that chromosome aneuploidy rates were occasionally skewed toward loss in both ImageStream and SCS. This is likely due to disruption of cytoplasmic micronuclei (MN) formed from lagging chromosomes during preparation for aneuploidy analysis (Crasta et al., 2012, Thompson and Compton, 2011) (Video S1; Figures S1C and S1D), as we observed fewer MN after preparation for ImageStream analysis (Figures S3B–S3D). There was no obvious enrichment of aneuploidy for chromosomes that were refractory to ImageStream analysis (chromosomes 4, 5, 13, 14, 19, 21, and 22) with SCS (Figure 1I), but we cannot exclude potential bias for these chromosomes below the limit of detection. Combining ImageStream analysis with SCS therefore demonstrates that specific chromosomes are prone to aneuploidy following the induction of chromosome mis-segregation using nocodazole washout, with chromosomes 1, 2, and 3 consistently affected.

Video S1. No Cell Death Is Observed within the 12 hr following Release from Nocodazole, Related to Figure S1 RPE1 cells stably expressing H2B-RFP were filmed following release from 8 h nocodazole treatment. A Quicktime movie of one field is shown. Stills from this movie are shown in Figure S1C.

### Chromosomes 1 and 2 Exhibit High Rates of Lagging at Anaphase in Multiple Non-transformed Cell Types

**Figure 2:**
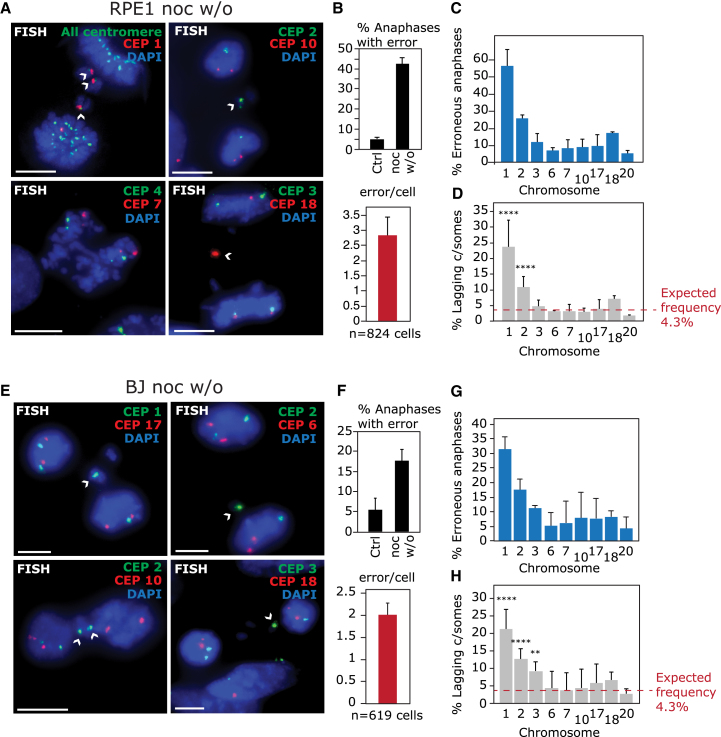
Chromosomes 1 and 2 Are Highly Prone to Lagging After Nocodazole Washout (A) RPE1 cells were treated with 8 hr nocodazole, then released for 1 hr before FISH with specific centromere enumeration probes as indicated. (B) Segregation error rates and average number of lagging chromosomes (errors) per erroneous anaphase. (C) Percentage erroneous RPE1 anaphases (one or more lagging chromosomes) exhibiting lagging of chromosomes indicated. (D) Quantification of percentage of lagging chromatids that are the chromosome indicated from erroneous anaphases. Total lagging chromatids were scored using DAPI-positive chromatid counting. Expected frequency is calculated using 1/23, assuming a random distribution among the 23 human chromosomes. (C) and (D) show mean ± SD of three independent experiments (except chromosome 17; two experiments), 268–481 lagging chromosomes analyzed per chromosome. (E) FISH of BJ cells after nocodazole treatment as in (A). (F) Segregation error rates and average number of errors per erroneous anaphase. (G) Percentage erroneous BJ anaphases exhibiting lagging of chromosomes indicated. (H) Quantification of percentage of lagging chromatids that are the chromosome indicated from erroneous anaphases (144–307 lagging chromosomes analyzed per chromosome). All experiments show mean ± SD of at least three independent experiments unless otherwise stated. ^∗∗^p < 0.005 and ^∗∗∗∗^p < 0.00005 (chi-square test; see Supplemental Experimental Procedures).

To examine whether chromosome-specific aneuploidy was reflected in chromosome lagging rates, nocodazole-treated RPE1 cells were released for 1 hr to observe anaphases (Figures S4A, S4B, 2A, and 2B). We performed FISH with specific centromere probes and determined the frequency of lagging of a panel of chromosomes. Strikingly, chromosomes 1 and 2 were found lagging in 56.4 ± 9% and 25.8 ± 2% of anaphases with errors (Figures 2A–2C) and constituted 23.3 ± 7% and 10.9 ± 3% of lagging chromatids, respectively, significantly higher than the 4.3% expected (p < 0.00005, chi-square test; Figure 2D). This indicates that more than a third of lagging chromatids following nocodazole washout are due to just two chromosomes and explains the consistently elevated aneuploidy of chromosomes 1 and 2. Aneuploidy rates in daughter cells are lower than lagging rates because merotelically attached lagging chromosomes are often resolved to the correct daughter cell (Cimini et al., 2004, Thompson and Compton, 2011). Nocodazole washout also enriched lagging of chromosomes 1 and 2 in BJ cells, primary human umbilical endothelial cells (HUVEC), and h-TERT-immortalized fallopian epithelial cells (FNE1) (Figures 2E–2H and S4C–S4J). These data demonstrate that chromosomes 1 and 2 are highly prone to chromosome mis-segregation following nocodazole washout, and this is common to multiple non-transformed cell types. Importantly, these data further establish the existence of biased chromosome mis-segregation by directly analyzing mitotic events before any selection effects can manifest. Chromosome 3, although detected as aneuploid in ImageStream and SCS, was prone to lagging in BJ cells but not RPE1 cells. This could be due to this chromosome’s becoming aneuploid through a mechanism other than lagging at anaphase or that we could not detect lagging of this chromosome at the time point analyzed in these cells. We therefore concentrated on understanding the molecular mechanism underlying the sensitivity of chromosomes 1 and 2 to mis-segregation following nocodazole washout.

### Chromosome 1 and 2 Lagging Is Not Dependent upon Kinetochore Expansion during Nocodazole Treatment

**Figure 3:**
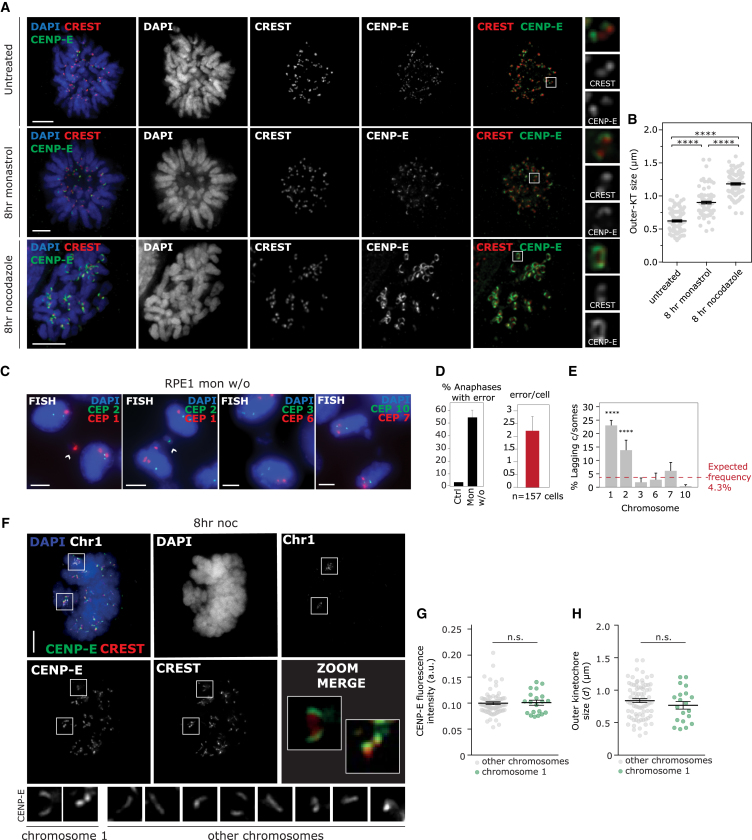
Biased Mis-segregation of Chromosomes 1 and 2 Is Not Dependent on Kinetochore Expansion (A) Immunofluorescence images of RPE1 cells treated with monastrol or nocodazole for 8 hr as indicated, stained with antibodies to mark centromeres (CREST serum, red) and outer kinetochores (CENP-E, green). (B) Kinetochore size quantification. (C) RPE1 cells were treated with 8 hr monastrol, then released for 1.5 hr before FISH with specific centromere enumeration probes as indicated. (D) Segregation error rates and average number of lagging chromosomes per erroneous anaphase. (E) Quantification of percentage of lagging chromatids that are the chromosome indicated from erroneous anaphases (77–299 lagging chromosomes analyzed per chromosome). (F) Immunofluorescence-FISH images of cells treated with nocodazole for 8 hr and stained with CREST sera, anti-CENP-E, and FISH using CEP1. (G and H) Quantification of outer kinetochore intensity (G) or expanded kinetochore size (H) at chromosome 1 compared with other chromosomes after 8 hr nocodazole. All experiments show mean ± SD of at least three independent experiments. ^∗∗^p < 0.005 and ^∗∗∗∗^p < 0.00005. See also Figure S5.

Nocodazole treatment abolishes microtubule (MT)-kinetochore attachments and leads to kinetochore expansion, the enlargement of the outer layer of the kinetochore (Hoffman et al., 2001, Thrower et al., 1996, Wynne and Funabiki, 2015). To test whether this phenomenon could explain biased mis-segregation, we induced chromosome mis-segregation in the absence of MT depolymerization. For this we inhibited Eg5 kinesis using monastrol, which prevents centrosome separation at prophase and thus leads to monopolar spindles. Upon drug washout, spindles reform in a manner that promotes merotelic attachment (Kapoor et al., 2000). Compared with nocodazole treatment, monastrol-treated cells displayed significantly lower kinetochore expansion as measured by CENP-E-marked outer kinetochore size (Figures 3A and 3B), in agreement with previous studies demonstrating that the majority of kinetochores remain attached syntelically to MTs upon Eg5 inhibition (Kapoor et al., 2000) and that expansion is not observed in *Xenopus* (Wynne and Funabiki, 2016) or human cells under these conditions (Sacristan et al., 2018). Monastrol washout treatment induced similar total lagging chromosome rates and also significantly enriched lagging of chromosomes 1 and 2 (Figures 3C–3E), suggesting that this bias is independent of extensive kinetochore expansion associated with nocodazole treatment. Furthermore, expanded kinetochores did not differ in size or intensity at chromosome 1 compared with other chromosomes after nocodazole treatment (Figures 3F–3H and S5). These data suggest that the enrichment of chromosome 1 and 2 lagging is unlikely to be due to chromosome-specific differences in kinetochore expansion.

### Chromosomes 1 and 2 Are Prone to Lagging following Mitotic Delay

**Figure 4:**
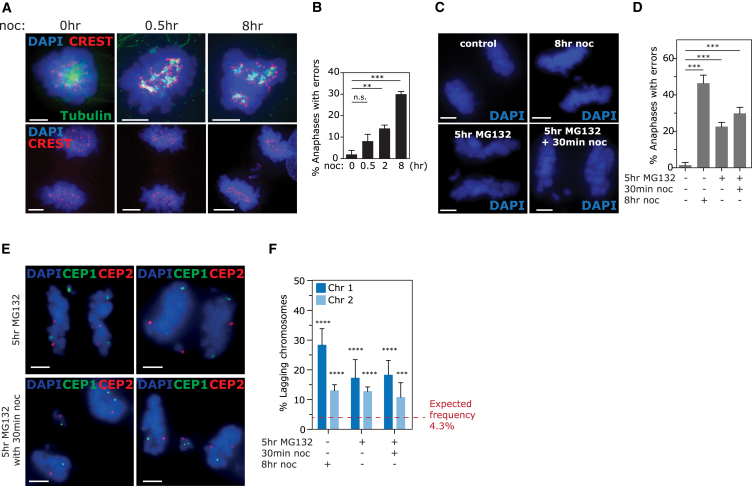
Chromosomes 1 and 2 Are Prone to Lagging following Mitotic Delay (A) Immunofluorescence of RPE1 cells treated with nocodazole for times indicated before fixing (top) or releasing for 1 hr, then fixing (bottom). (B) Quantification of anaphase lagging rates from (A). (C) Immunofluorescence images of cells treated as indicated. (D) Quantification of anaphase lagging rates from (C). (E) Cells were treated as in (C) and (D) before FISH with centromere enumeration probes as indicated. (F) Quantification of percentage lagging chromosomes (113–298 total lagging chromosomes analyzed) that are chromosomes 1 and 2. All experiments show mean ± SD of at least three independent experiments. ^∗∗∗^p < 0.0005 and ^∗∗∗∗^p < 0.00005 (chi-square test).

We then asked which aspect of nocodazole or monastrol treatment was responsible for elevated lagging of chromosomes 1 and 2. Both treatments include passage through abnormal spindle geometry intermediates and a period of mitotic delay, commonly used to elevate the number of anaphase cells available for analysis. To dissect the relative contributions of mitotic delay and abnormal spindle geometry, we set out to analyze specific chromosome lagging rates after abnormal spindle formation but in the absence of mitotic delay. We treated cells with the minimum nocodazole treatment period required to fully disassemble all MTs (30 min; Figure S6A) before washout. Interestingly segregation error rates increased only slightly (from 1.9 ± 3% to 8.1 ± 6%; Figures 4A and 4B), resulting in too few lagging chromosomes to allow accurate analysis of biased mis-segregation. In fact, we noticed a linear relationship between time spent in nocodazole-induced prometaphase and total lagging chromosome rates (Figure 4B). This was not due to incomplete MT depolymerization, as mitotic cells displayed efficient loss of MTs after all nocodazole treatment times (Figures 4A and S6B). This was also not due to fewer cells affected by nocodazole, as live-cell imaging of prometaphase cells released from nocodazole-induced mitotic arrest exhibited the same relationship between length of nocodazole treatment and rate of segregation errors (Figures S6C and S6D). A similar phenomenon was also observed following Eg5 inhibition and release (Figures S6E and S6G). These findings suggested that mitotic delay during nocodazole or monastrol treatment is an important cause of chromosome mis-segregation. To test this, we induced mitotic delay in the absence of spindle defects by treating cells with the proteasome inhibitor MG132 to prevent anaphase onset. Prolonged treatment with MG132 can lead to multipolar spindles and premature sister chromatid separation that irreversibly activates the mitotic checkpoint (Daum et al., 2011, Lara-Gonzalez and Taylor, 2012). To circumvent this, we limited MG132 treatment to 5 hr before washout and only analyzed lagging chromosomes from bipolar anaphases. Interestingly, this treatment significantly elevated chromosome segregation errors compared with control cells (from 1.3 ± 1.5% to 22.5 ± 2.4%; Figures 4C and 4D). The addition of a brief treatment with nocodazole before MG132 washout slightly increased the error rate (from 22.5 ± 2.4% to 29.75 ± 3.4%; Figure 4D). This suggests that both abnormal spindle formation and mitotic delay contribute to promote anaphase lagging. We then analyzed chromosome-specific lagging rates and observed that MG132-induced mitotic delay was sufficient to significantly enrich lagging of chromosomes 1 and 2 (Figures 4E and 4F). Taken together, these data suggest that mitotic delay is a major contributor to mis-segregation induced by nocodazole or Eg5 inhibitor washout treatments and that this delay itself introduces the bias for chromosome 1 and 2 lagging.

### Cohesion Fatigue Contributes to Mitotic Delay-Induced Chromosome Mis-segregation

**Figure 5:**
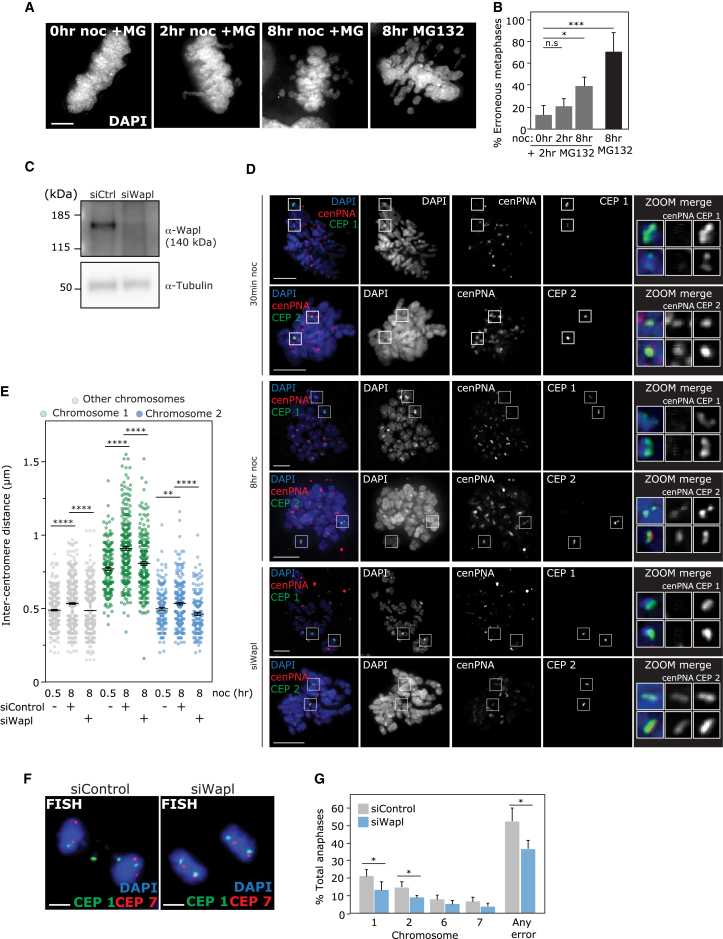
Cohesion Fatigue Contributes to Mitotic Delay-Induced Chromosome Mis-segregation (A and B) Representative images (A) and quantification (B) of RPE cells that were treated with nocodazole as indicated then released into MG132 for 2 hr, or treated with MG132 for 8 hr, before scoring percentage of cells with unaligned chromosomes. (C) RPE1 cells were treated with small interfering RNA (siRNA) (non-targeting or against Wapl) for 48 hr before western blotting with Wapl antibody (alpha-tubulin used as loading control). (D and E) Representative images (D) and quantification (E) of RPE cells that were treated with siRNA (non-targeting or against Wapl) before treatment with 8 hr nocodazole (48 hr siRNA in total), then FISH using PNA (peptide nucleic acid) centromere-targeted probes (red) and specific centromere probes as indicated in green. Note that no PNA signal was visible at centromere 1, so these measurements were made using the centromere-specific probe signal. (F) RPE1 cells were treated with siRNA (non-targeting or against Wapl) for 39 hr before 8 hr nocodazole, washout for 1 hr (48 hr siRNA in total), then FISH with centromere probes as indicated. (G) Percentage total anaphases with errors in any chromosome or specific chromosomes were analyzed as indicated. All experiments show mean ± SD of at least three experiments. See also Figure S7.

A known consequence of delay in mitosis is gradual failure of the cohesive force holding sister chromatids together, “cohesion fatigue,” that can lead to premature sister chromatid separation (PSCS) (Daum et al., 2011, Manning et al., 2010, Stevens et al., 2011, van Harn et al., 2010). These studies suggested that MT pulling forces are required for cohesion fatigue. However, it has also been shown that increasing prometaphase delay in the absence of bipolar kinetochore attachment in INCENP-variant cells can increase rates of subsequent PSCS following re-establishment of a bipolar spindle (Hengeveld et al., 2017). To test whether our nocodazole treatment conditions could prime chromosomes for subsequent cohesion fatigue, we treated cells with nocodazole for increasing time before washout into MG132, to allow chromosome-MT attachments to form but prevent anaphase onset. Pre-treatment with 8 hr of nocodazole led to a significant increase in metaphases with scattered chromosomes indicating PSCS (Daum et al., 2011, Stevens et al., 2011) (Figures 5A and 5B). Scattering was increased further in cells treated with MG132 alone for 8 hr, in agreement with previous studies demonstrating that dynamic MTs during the arrest period are required for maximal PSCS (Daum et al., 2011, Stevens et al., 2011). We next tested whether cohesion fatigue was a factor in mitotic delay-induced chromosome mis-segregation. We depleted the negative regulator of cohesion Wapl (Gandhi et al., 2006, Kueng et al., 2006) using RNAi (Figure 5C) to enhance the stability of cohesion on DNA. This was shown previously to reduce rates of chromosome scattering at metaphase (Daum et al., 2011, Lara-Gonzalez and Taylor, 2012, Stevens et al., 2011). Increased inter-centromere distance is a marker for reduced cohesion (Manning et al., 2010). Accordingly Wapl depletion rescued elevated inter-centromere distances caused by 8 hr nocodazole treatment at centromeres generally and at chromosomes 1 and 2 (Figures 5D and 5E). Wapl depletion also significantly reduced rates of anaphase lagging caused by nocodazole washout both globally and of chromosomes 1 and 2 (Figures 5F and 5G). Wapl depletion did not fully rescue lagging rates, potentially because of MT pulling forces counteracting the protection from siWapl. It is also possible that additional mechanisms operate alongside cohesion fatigue to drive biased mis-segregation of chromosomes 1 and 2. Mitotic delay induced by nocodazole, Eg5 inhibitors, or MG132 therefore leads to a deterioration of centromeric cohesion and a concomitant increase in chromosome lagging that can be partially counteracted by increasing the stability of cohesion on DNA.

### Chromosomes 1 and 2 Are Particularly Prone to Cohesion Fatigue

**Figure 6:**
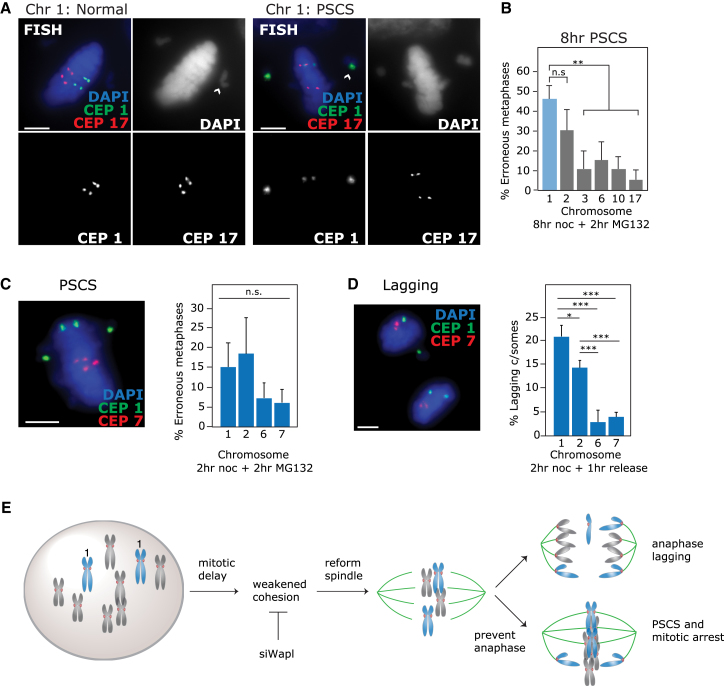
Chromosomes 1 and 2 Are Particularly Prone to Cohesion Fatigue (A and B) Representative images (A) and quantification (B) of RPE cells that were treated with 8 hr nocodazole, then 2 hr MG132 before FISH with specific centromere enumeration probes (CEPs) and quantification of PSCS for each chromosome indicated. Erroneous metaphases (one or more unaligned chromosomes) exhibiting PSCS of a panel of chromosomes was quantified. (C) RPE1 cells were treated with 2 hr nocodazole, then 2 hr MG132 before FISH with centromere-specific probes as indicated and quantification of PSCS for each chromosome indicated. (D) RPE1 cells were treated with 2 hr nocodazole, then released for 1 hr before FISH with specific centromere enumeration probes and scoring lagging chromosomes as indicated. All experiments show mean ± SD of three independent experiments. (E) Model to explain the behavior of chromosomes 1 and 2 during mitotic arrest. Chromosomes 1 and 2 are prone to cohesion fatigue that can manifest as (1) propensity to lagging at anaphase and resulting aneuploidy in daughter cells or (2) premature sister chromatid separation (PSCS) leading to irreversible mitotic arrest.

Next, we tested the predisposition of individual chromosomes to cohesion fatigue following nocodazole treatment by analyzing chromosome-specific rates of PSCS in metaphases that displayed chromosome scattering. Strikingly, chromosomes 1 and 2 were particularly prone to PSCS after 8 hr nocodazole pre-treatment compared with other chromosomes (Figures 6A and 6B). Additionally metaphase spreads revealed greater inter-centromere distance at chromosome 1 compared with chromosome 6, which increased with longer treatment with nocodazole, and a higher incidence of separated chromosome 1 sister chromatids following 8 hr nocodazole (Figures S7A and S7B). Cohesion fatigue has been observed after only short periods of mitotic arrest (Daum et al., 2011, Stevens et al., 2011). Accordingly, despite lower absolute rates of chromosome scattering and lagging (Figures 4B and 5B), bias toward chromosomes 1 and 2 was evident in both PSCS and anaphase lagging after only 2 hr nocodazole washout (Figures 6C and 6D). Importantly, this demonstrates that enriched lagging of chromosomes 1 and 2 is promoted by even brief periods of mitotic arrest that could be relevant in cancer cells (Potapova and Gorbsky, 2017). Taken together, these data suggest that mitotic delay leads to weakened cohesion that (1) cannot resist MT pulling forces upon subsequent metaphase delay, (2) promotes incorrect kinetochore-MT attachment and anaphase lagging, and (3) particularly affects chromosomes 1 and 2 (see model in Figure 6E).

### Different Mechanisms Promoting Mis-segregation Induce Distinct Biases

**Figure 7:**
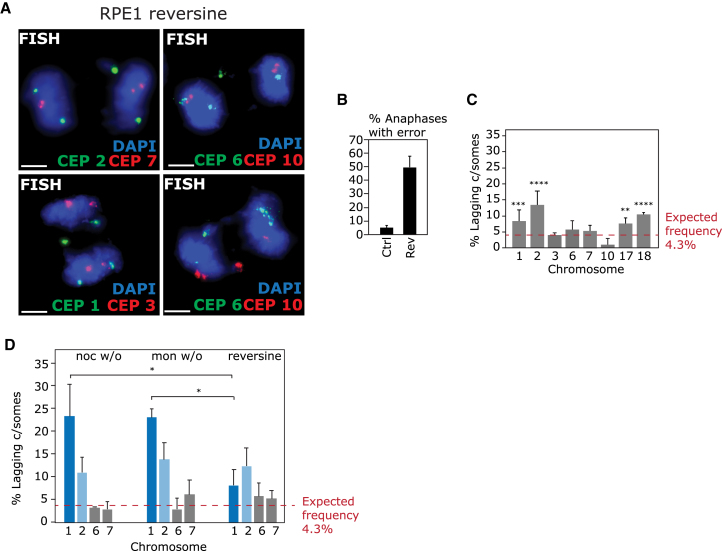
Reversine Treatment Induces Different Biases (A) RPE1 cells were treated with 250 nM reversine for 5 hr to induce lagging chromosomes before FISH with centromeric probes as indicated. (B) Percentage anaphases with lagging chromosomes was quantified. (C) Quantification of percentage of lagging chromatids (122–612 errors per chromosome analyzed) that are the chromosome indicated from erroneous anaphases. All experiments show mean ± SD of three experiments. (D) Summary graph of conditions collated from Figures 2, 4, and 7. ^∗^p < 0.05.

Finally, we assessed whether inducing chromosome mis-segregation by a different means would also lead to biased mis-segregation. We treated cells with reversine, a small-molecule inhibitor of the mitotic checkpoint kinase Mps1 that promotes chromosome mis-segregation through impairing correct outer kinetochore regulation and simultaneously disrupting mitotic checkpoint signaling (Santaguida et al., 2010). This treatment induced similar overall lagging chromosome rates compared with nocodazole washout (Figures 7A and 7B), but the pattern of bias was different from that observed following nocodazole or monastrol washout; Chromosome 1 lagging was significantly reduced (Figures 7C and 7D), and chromosomes 17 and 18 were now significantly enriched (Figure 7C). These data suggest that different methods to induce chromosome mis-segregation generate different biases, which could reflect either differences in the nature of lagging chromosomes produced (e.g., unattached or merotelic) or different mechanistic origins of kinetochore mal-attachment between these conditions.

## Discussion

We demonstrate that chromosome mis-segregation and aneuploidy are non-randomly distributed among human chromosomes following induction of aneuploidy using drug-induced mitotic delay and Mps1 inhibition. Treatments that induce mitotic delay lead to cohesion fatigue and anaphase lagging and a bias for chromosomes 1 and 2, even in the absence of spindle defects. We also show that chromosomes 1 and 2 are particularly vulnerable to cohesion fatigue, suggesting that an inherent susceptibility to cohesion fatigue may contribute to biased mis-segregation and aneuploidy observed following nocodazole washout. This insight into the mechanisms and bias of chromosome mis-segregation caused by mitotic delay is particularly relevant for studies using nocodazole or monastrol washouts to induce chromosome mis-segregation and also has clinical relevance because of the widespread use of common cancer chemotherapeutics such as vincristine and paclitaxel, which arrest cells in mitosis for prolonged periods.

### Cohesion Fatigue Induced by Mitotic Delay Promotes Chromosome Mis-segregation and Specifically Affects Chromosomes 1 and 2

The mechanisms linking mitotic delay, cohesion fatigue, and anaphase lagging are poorly understood. Cohesion fatigue could elevate chromosome mis-segregation because of effects on centromeric geometry or flexibility that might increase merotelic attachment rate (Sakuno et al., 2009). It has also been suggested that stretched inter-kinetochore distance seen in mild cohesion fatigue (i.e., before complete PSCS) could displace high inner centromeric aurora B, leading to increased incidence of merotelic attachment (Sapkota et al., 2017). Alternatively, because multiple studies have demonstrated an intricate interplay between chromosome cohesion factors and regulation of the chromosomal passenger complex (CPC), responsible for error correction (reviewed in Trivedi and Stukenberg, 2016, Mirkovic and Oliveira, 2017, Kleyman et al., 2014), it is possible that cohesion fatigue might prevent efficient correction of mal-attachments by improper regulation of the CPC. A key remaining question is what features of centromeres at chromosomes 1 and 2 explain their propensity to undergo cohesion fatigue. It is possible that differences in centromeric composition underlie this sensitivity. Of note, large regions of pericentric heterochromatin have been identified at the q arms of chromosomes 1, 3, 4, 9, 16, and 19 (Atkin and Brito-Babapulle, 1981, Craig-Holmes and Shaw, 1971) (Figure 1A), although it is not clear whether the nature of chromosome 1 pericentric heterochromatin differs qualitatively and how this might render chromosomes prone to cohesion fatigue.

### Features Underlying Bias in Mis-segregation Rates

Our data suggest that the propensity of chromosomes 1 and 2 to undergo cohesion fatigue contributes to their biased mis-segregation, but other mechanisms could also contribute. Chromosomes 1 and 2 are the largest chromosomes in humans (Figure 1A). One idea is that longer chromosomes might require a “stronger” centromere and that centromere length or size may need to scale functionally with chromosome length. However, it has been suggested that drag produced by chromosomes is negligible in comparison with spindle forces (Civelekoglu-Scholey and Scholey, 2010, Nicklas, 1983), so larger chromosomes do not necessarily possess a requirement for a stronger centromere. Indeed, centromere size does not scale with chromosome length in humans (Table S1). Moreover we did not observe any differences in outer kinetochore structure measured by CENP-E intensity between chromosome 1 and other chromosomes following nocodazole treatment and associated kinetochore expansion (Figure 3). Nevertheless a correlation has been observed between chromosome size and levels of the inner centromeric protein CENP-A in human cells (Irvine et al., 2004), suggesting that kinetochore size or function may vary between chromosomes. In this regard, it is also interesting that chromosome 18, with the longest alpha satellite length (5.4 Mb; Table S1) was significantly enriched in lagging chromosomes following reversine treatment and exhibited moderate but consistent effects in response to nocodazole washout both in terms of ImageStream aneuploidy and anaphase lagging analyses, despite falling short of statistical significance. This suggests that centromere size could in fact contribute to biased mis-segregation under certain conditions. Accordingly, it has recently been shown in Indian Muntjak cells that increased centromere size predisposes to merotelic attachment (Drpic et al., 2018). An alternative possibility is that larger chromosomes may be prone to mis-segregation because of their tendency to occupy peripheral positions that might predispose to merotelic attachment (Cimini et al., 2004, Khodjakov and Rieder, 1996).

### Potential Role of Non-random Chromosome Mis-segregation in the Development of Cancer Aneuploidy Landscapes

Merotelic attachment and cohesion defects have both been proposed to contribute to cancer CIN (Bakhoum et al., 2009, Brownlee et al., 2014, Ertych et al., 2014, Kawasumi et al., 2017, Manning et al., 2014, Solomon et al., 2014). However, confirming whether specific chromosomes are prone to mis-segregation during tumorigenesis is non-trivial. The bulk of available tumor genomic information lacks single cell resolution and is heavily shaped by evolutionary selection processes (Greaves and Maley, 2012, McGranahan and Swanton, 2017) that might obscure signatures of non-random mis-segregation. Nevertheless, this phenomenon could influence early events during tumorigenesis. For example, lagging chromosomes can be subject to downstream DNA damage events such as breakage-fusion-bridge events and chromothripsis that could fuel subsequent structural aneuploidy events (Crasta et al., 2012, Janssen et al., 2011, Zhang et al., 2015). In this regard, it is interesting that chromosomes 1 and 2 are among the three chromosomes most frequently affected by copy number alteration in primary retinoblastomas (Kooi et al., 2016), and are frequently affected by incorporation into MN and resulting chromothripsis following nocodazole washout (Zhang et al., 2015). Given links between dysfunction of the retinoblastoma protein pRB, cohesion defects and chromosome lagging (Manning et al., 2010, Manning et al., 2014), and the propensity for chromosomes 1 and 2 to lag under conditions of mal-attachment and cohesion fatigue, it is possible that non-random mis-segregation could act in concert with evolutionary selection to drive these recurrent SCNA patterns in retinoblastomas and could potentially act more broadly across additional cancer types.

## Experimental Procedures

### Cell Culture and RNAi

All cell lines were maintained at 37°C with 5% CO_2_ (see Supplemental Experimental Procedures for details of origin and media). hTERT-RPE-1 H2B-RFP stable cell lines were generated after transfection with lentiviral construct H2B-RFP (26001; Addgene). RNAi was achieved by transfection of cells for 48 hr with 30 nM small interfering RNA (siControl [D-001210-02] and siWAPL SMART pool [M-026287-01]; Dharmacon) using Lipofectamine RNAiMAX (Invitrogen) and Optimem (Gibco). Drug concentrations used were 10 μM MG132, 100 ng/mL nocodazole, 10 μM S-trityl-L-cysteine (STLC), 100 μM monastrol, and 250 nM reversine (all from Sigma-Aldrich). Release from mitotic arrest was achieved by washing drug out of cells with prewarmed media three to five times, then leaving in incubator for 1 hr (nocodazole), 1.5 hr (STLC and monastrol), or 2.5 hr (MG132).

### Apoptosis Assay, Trypan Blue Viability, and Cell Cycle Analysis

Cells were re-plated after either only trypsinization or after 8 hr nocodazole treatment followed by mitotic shake-off. After 12 hr, cells were collected and then either (1) stained with annexin V Alexa Fluor 647 antibody (A23204; Thermo Fisher Scientific) and DAPI, fixed in 1% formaldehyde and analyzed using BD FACS Diva 8.2, or (2) fixed in 4% formaldehyde for 7 min, then permeabilized with 0.2% Triton X-100 for 2 min, stained with DAPI, and analyzed using BD FACS Diva 8.2. Cell cycle profiles were quantified using FlowJo. For viability assay, re-plated cells at indicated time points were stained with trypan blue (Gibco), and percentage cell death was calculated using TC20 Automated Cell Counter (Bio-Rad).

### Immunoblotting

Cell lysates were prepared by a lysis buffer (20 mM Tris-HCl [pH 7.4], 135 mM NaCl, 1.5 mM MgCl_2_, Triton 1%, glycerol 10%, and 1× protease inhibitor [Roche]). Immunoblots were probed with antibodies against Wapl (Sc-365189; Santa Cruz) and alpha-tubulin (T0674; Sigma-Aldrich) and developed using goat anti-mouse IgG horseradish peroxidase (HRP) conjugated antibody (Sc-2005; Santa Cruz) in a Chemidoc (GE Healthcare).

### Immunofluorescence

Cells grown on glass slides or coverslips were fixed with PTEMF (0.2% Triton X-100, 0.02 M PIPES [pH 6.8], 0.01 M EGTA, 1 mM MgCl_2_, and 4% formaldehyde). After blocking with 3% BSA, cells were incubated with primary antibodies according to suppliers’ instructions: beta-tubulin (ab6046; Abcam), Centrin 3 (ab54531; Abcam), CREST (15-234-0001; Antibodies Incorporated), and CENP-E (ab5093; Abcam). Secondary antibodies used were goat anti-mouse Alexa Fluor 488 (A11017; Invitrogen), goat anti-rabbit AF594 and AF488 (A11012 and A11008; Invitrogen), and goat anti-human AF647 (109-606-088-JIR [Stratech] or A21445 [Invitrogen]). DNA was stained with DAPI (Roche), and coverslips were mounted in Vectashield (Vector H-1000; Vector Laboratories).

### Metaphase Spreads

Cells collected from mitotic shake-off were re-suspended in 75 mM KCl hypotonic solution for 30 min at 37°C. Cells were pelleted and re-suspended in freshly prepared 3:1 methanol-glacial acetic acid, then dropped onto slides.

### Fluorescence *In Situ* Hybridization

Cells were grown on glass slides, fixed in methanol/acetic acid, then put through an ethanol dehydration series. Cells were incubated overnight at 37°C with specific centromere enumeration probes (CEP) (Cytocell) or pan-centromere probes (Cambio), then washed the following day with 0.25× saline sodium citrate (SSC) at 72°C followed by 2× SSC and 0.05% Tween. When measuring cohesion fatigue, PSCS was defined as either one or both centromere signals of one sister chromatid pair completely separated from the metaphase plate.

### FISH with PNA Centromere Probe

Metaphase spreads were prepared as above, and peptide nucleic acid (PNA) staining was achieved following the manufacturer’s instructions (Eurogentec). In brief, slides were washed in PBS at 37°C and fixed in 4% formaldehyde in PBS. After fixation, cells were dehydrated with an ethanol series and air-dried. Cells and PNA centromere probe were denatured for 15 min at 85°C, incubated for 1 hr at room temperature, then washed with 2× SSC and 0.01% Tween at 60°C.

### IF-FISH

Mitotic cells were collected and re-suspended in 75 mM KCl hypotonic solution for 30 min on ice. Then cells were pelleted, re-suspended in freshly prepared PTEMF solution, and dropped onto slides. Immunofluorescence (IF) and FISH were performed as above, with the addition of washes with 100 mM Tris-HCl, 150 mM NaCl, and 0.5% BSA between primary and secondary antibodies in the IF procedure.

### ImageStream FISH and Analysis

ImageStream FISH was performed in suspension: cells in log phase growth were treated with 100 ng/mL nocodazole for 8 hr and released following mitotic shake-off into fresh medium for 12 hr. Cells were fixed by adding freshly prepared 3:1 methanol-glacial acetic acid dropwise to a pellet of PBS-washed cells. For hybridization, cells were washed with 1× PBS with 3% BSA twice for 5 min, pelleted, and resuspended in 0.05% Tween 20 and 2× SSC in PBS. One million cells were pelleted and resuspended in complete hybridization mixture: 28 μL hybridization buffer, 10 μL nuclease-free H_2_O, and 2 μL CEP probe. Denaturing and probe hybridization were performed in a thermocycler under the following conditions: 80°C (5 min), 42°C (9–16 hr), and an optional storage step of 4°C. Following hybridization, 200 μL of 2× SSC and 0.05% Tween was added to each reaction mixture. Cells were pelleted and resuspended in 50–100 μL of 1× PBS before analysis (optional: DAPI, 1 μg/mL). See Supplemental Experimental Procedures for details of ImageStream analysis.

### Microscopy

Images were acquired using an Olympus DeltaVision RT microscope (Applied Precision) equipped with a Coolsnap HQ camera. Three-dimensional image stacks were acquired in 0.2 μm steps, using Olympus 100× (1.4 numerical aperture), 60×, or 40× UPlanSApo oil immersion objectives. Deconvolution of image stacks and quantitative measurements was performed with SoftWorx Explorer (Applied Precision). H2B-RFP-labeled cells were live imaged in a four-well imaging dish (Greiner Bio-one). Twenty micrometer z stacks (10 images) were acquired using an Olympus 40× 1.3 numerical aperture UPlanSApo oil immersion objective every 3 min for 8 hr using a DeltaVision microscope in a temperature and CO_2_-controlled chamber. Analysis was performed using Softworx Explorer. To observe cell death after nocodazole washout, cells were imaged every 3 min for the first 4 hr and then every 15 min for another 8 hr.

### Kinetochore Intensity and Size Measurements

Outer-kinetochore size measurement was performed with SoftWorx Explorer (Applied Precision), using the measure tool to measure the maximum outer distance between CENP-E signals at centromeres as determined by CREST signal. CENP-E fluorescence mean intensity was measured within 1 μm spheres centered around CREST-marked centromeres, using IMARIS (BITPLANE). Measurements were obtained from ten kinetochores per cell using projections of ten 0.2 μm z stacks and ten cells per condition.

### Single-Cell Sequencing

Samples from control and experimentally induced aneuploid cells were sorted by FACS prior to next-generation sequencing library preparation and data analysis using AneuFinder as previously reported (Bakker et al., 2016, van den Bos et al., 2016). See Supplemental Experimental Procedures for further details.

### Statistical Analysis

Unpaired t test, one-way ANOVA with post hoc Tukey’s comparison, or chi-square tests were used to test for levels of significance using either Excel (Microsoft), Prism (GraphPad), or MATLAB (R2016B; The MathWorks) (see Supplemental Experimental Procedures).

## Data and Software Availability

The accession number for the raw single-cell sequencing reads is European Nucleotide Archive: PRJEB25738.

## Supplemental Information

Document S1. Supplemental Experimental Procedures, Figures S1–S7, and Tables S1 and S2

Document S2. Article plus Supplemental Information
