# Supplementary material for: Non-random Mis-segregation of Human Chromosomes
Source: Cell Rep. 2018 Jun 13;23(11):3366–80. doi: 10.1016/j.celrep.2018.05.047 (PMC6019738; doi:10.1016/j.celrep.2018.05.047)
Supplement: Document S1. Supplemental Experimental Procedures, Figures S1–S7, and Tables S1 and S2 [file mmc1.pdf]

Cell Reports, Volume *23*

Supplemental Information

Non-random Mis-segregation of Human Chromosomes

Joseph Thomas Worrall, Naoka Tamura, Alice Mazzagatti, Nadeem Shaikh, Tineke van Lingen, Bjorn Bakker, Diana Carolina Johanna Spierings, Elina Vladimirou, Floris Foijer, and Sarah Elizabeth McClelland

Supplemental Figure 1 (relating to Figure 1)

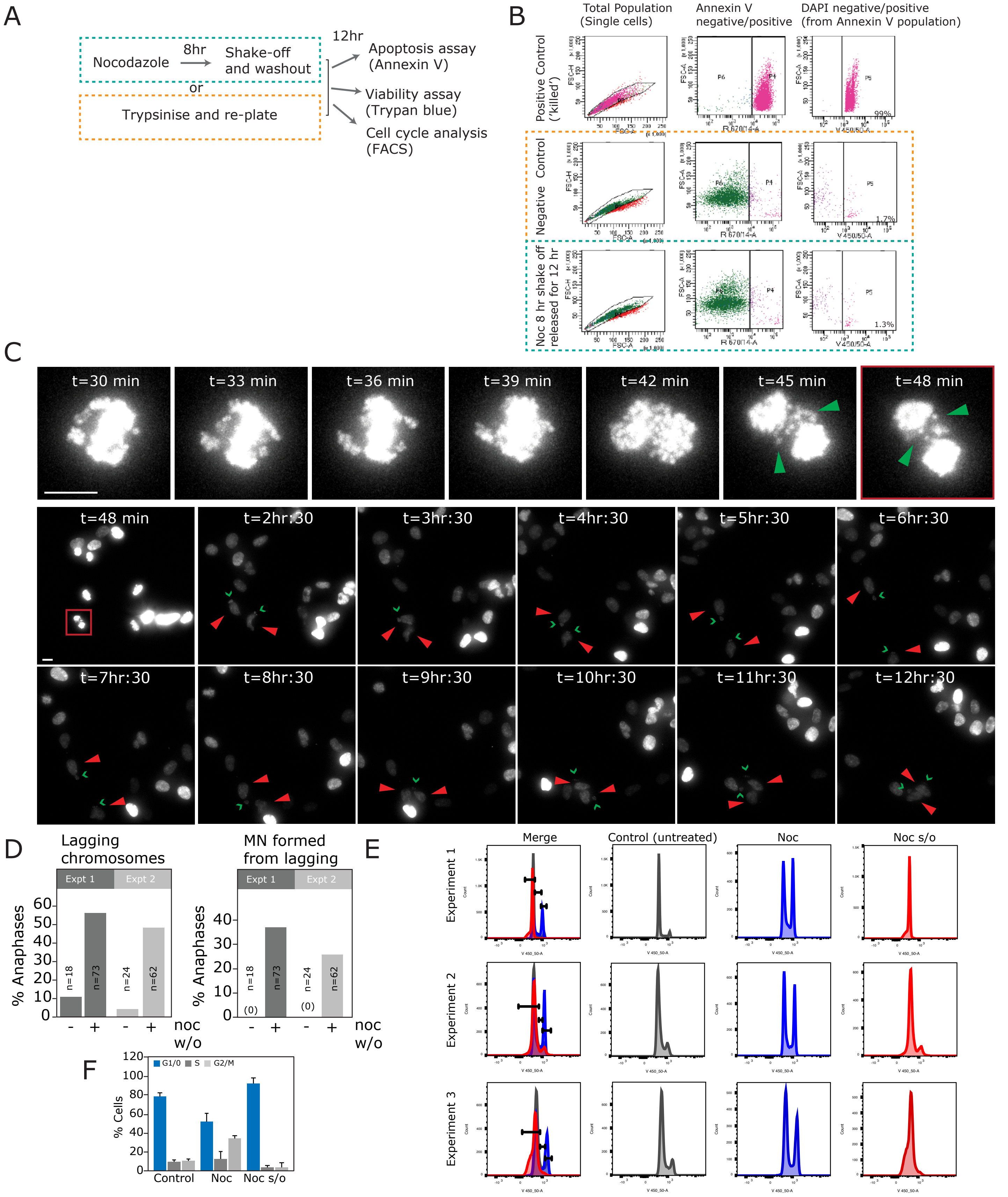

Figure S1 (relating to Figure 1). Aneuploidy-mediated cell death does not occur prior to ImageStream analysis. (**A**) Schematic indicating experimental workflow for experiments in (B,E,F). (**B**) Flow cytometry plots of cells subjected to Annexin V apoptosis assay. Positive 'killed' control cells (top panels) were fixed prior to analysis to induce apoptosis and provide the gating for the untreated and nocodazole washout treated cells (middle and lower panels). (**C**) RPE1 cells stably expressing H2B-RFP were filmed following release from 8 hr nocodazole. Filming began 30 min after drug washout and cells were imaged every 3 min for 4 hr, then every 15 min for a further 8 hr (12 hr total filming). Stills from Supplementary Movie 1 are shown. Green arrowhead indicates an anaphase cell with lagging chromosomes and chevrons indicate micronuclei formed from the lagging chromosomes. Red arrowheads mark daughter cells throughout the remainder of the movie. 39 daughter cells from mothers exhibiting lagging chromosomes could be followed for the full 12 hr (cells frequently move 'off screen' during the subsequent hours) with none exhibiting cell death. Cell death rates of all cells from movies are given in Figure 1. Scale bars 10 μM. (**D**) Segregation error rates and anaphases with micronuclei resulting from lagging chromosomes were quantified from the first 4 hr of imaging from two independent movies. (**E, F**) Flow cytometry plots (E) and quantification (F) of RPE1 cells subjected to cell cycle analysis in control, 8 hr nocodazole or 8 hr nocodazole followed by shake-off (s/o) and 12 hr release, using DAPI staining and quantification using FlowJo. Mean and SD from three independent experiments are shown in F.

Supplemental Figure 2 (relating to Figure 1)

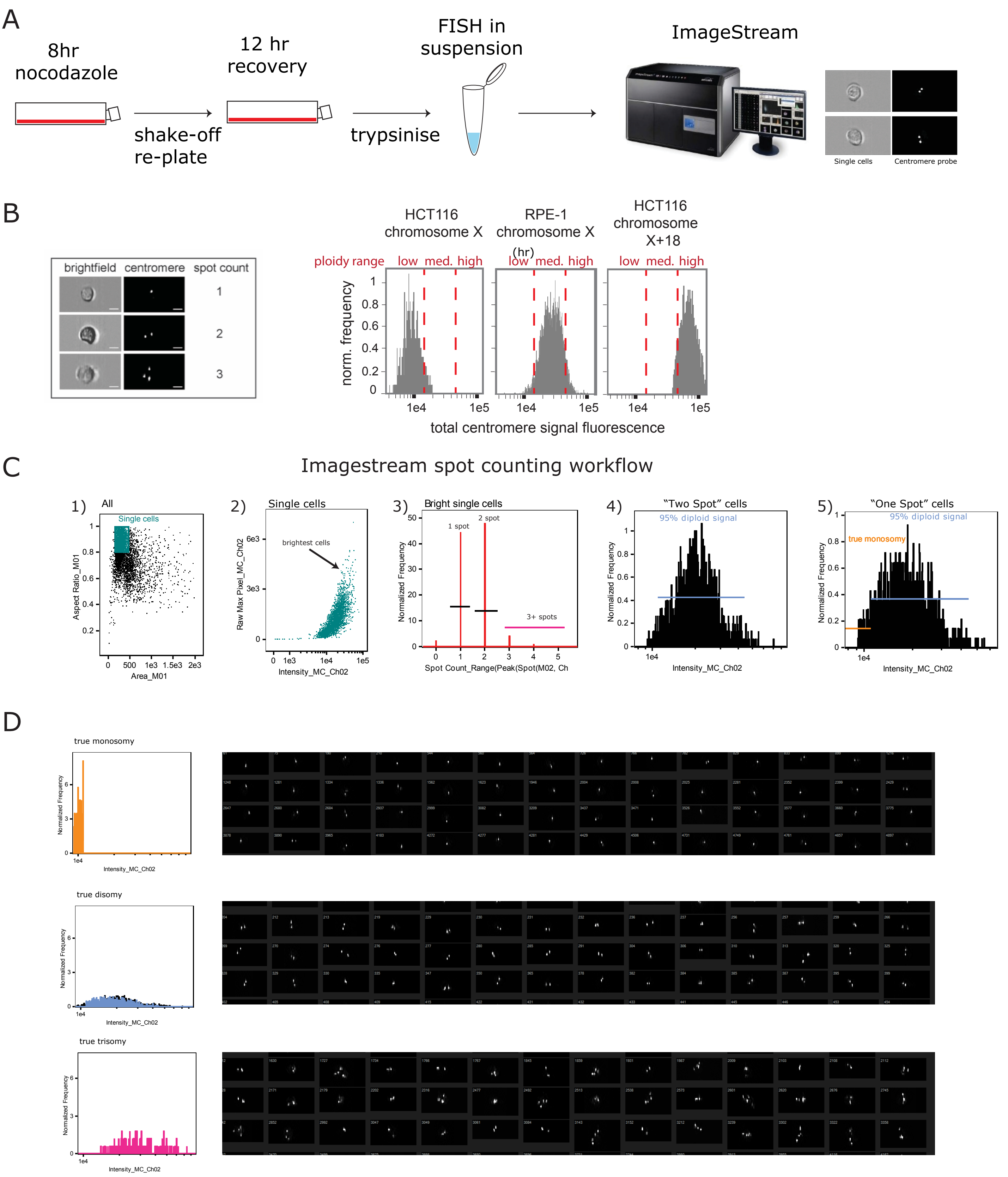

Figure S2 (relating to Figure 1). ImageStream methodology. (A) Schematic indicating experimental workflow prior to ImageStream analysis. (B) Examples of cells stained with FISH probes, imaged and analysed by Imagestream. HCT116 cells probed with CEP X (only one copy present, used to calibrate for monosomy), RPE-1 cells probed with CEP X (two copies present to calibrate disomy), HCT116 cells probed with both CEP X and CEP 18 (generates three foci in total to calibrate for detecting trisomy). Histograms of foci intensities for these cell populations shown to the right. (C) Imagestream workflow (method adapated from Minderman et al., 2012). (1) Gate for single cells in total population. (2) Raw max pixel (brightest focus in cell) should increase as total intensity of cell increases, to indicate high specificity of staining of foci. (3) Imagestream counts how many cells have 0,1,2 or 3+ foci and produces histogram. (4) Assuming foci intensities of cells in diploid population is normally distributed, select central 95% range. (5) Find this range of intensities in the monosomy population. Any "monosomy" cell in this focus intensity range is probably a diploid cell with two spots overlapping into a single, overly bright spot. We therefore move that population of cells up to the diploid population. Any cells left that are less bright are now classed as true monosomy cells. (D) Representative images and corrected intensity histograms of monosomic, disomic and trisomic populations respectively.

Supplemental Figure 3 (relating to Figure 1)

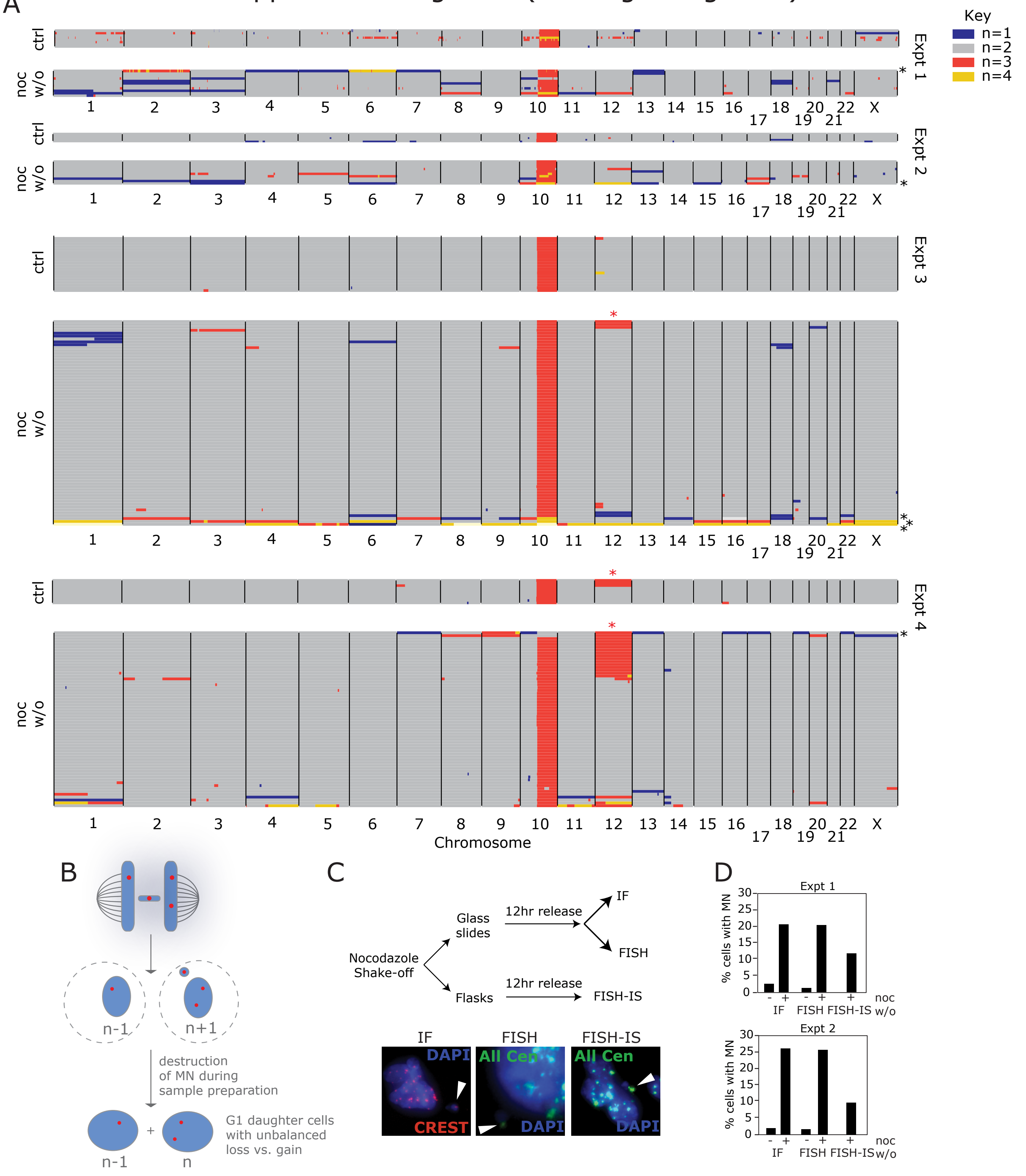

Figure S3 (relating to Figure 1). Single cell sequencing corroborates ImageStream aneuploidy of chromosomes 1-3 and micronuclei are prone to destruction during ImageStream preparation. (**A**) Genome-wide copy number profiles of control, and nocodazole washout treated RPE1 cells from single cell sequencing data analysed using AneuFinder (Bakker et al., 2016) (four independent experiments; 44 control and 144 nocodazole w/o cells in total). Each row represents a single cell with chromosomes plotted as columns. Copy number states are depicted in different colours (see key). Cells with more than 6 aneuploidies per cell were discounted from the analysis as this suggested a multipolar division (6 cells were removed under these criteria; black asterisks). Gains of chromosome 12 were also discounted due to the known low levels of trisomy 12 in this cell line (red asterisks) (Di Nicolantonio et al., 2008; Zhang et al., 2015). Known amplification of chromosome 10q is caused by an unbalanced translocation to the X chromosome. (**B**) Cartoon illustrating the possible fate of a lagging chromosome that is incorporated into a micronucleus that is then lost from daughter cells. This leads a net loss of chromosome copy number in daughter cells. (**C**) Schematic of experimental workflow to determine the effect of preparation for FISH either on microscopy slides ('FISH') or FISH-in-suspension ('FISH-IS') on micronucleus detection rates. Representative images are shown of cells fixed for immunofluorescence and stained with CREST anti-sera, fixed for FISH and probed with pan-centromere probe, or prepared for ImageStream analysis, followed by pipetting onto glass slides (see Methods). (**D**) Quantification of MN rate from conditions as indicated in (C).

Supplemental Figure 4 (relating to Figure 2)

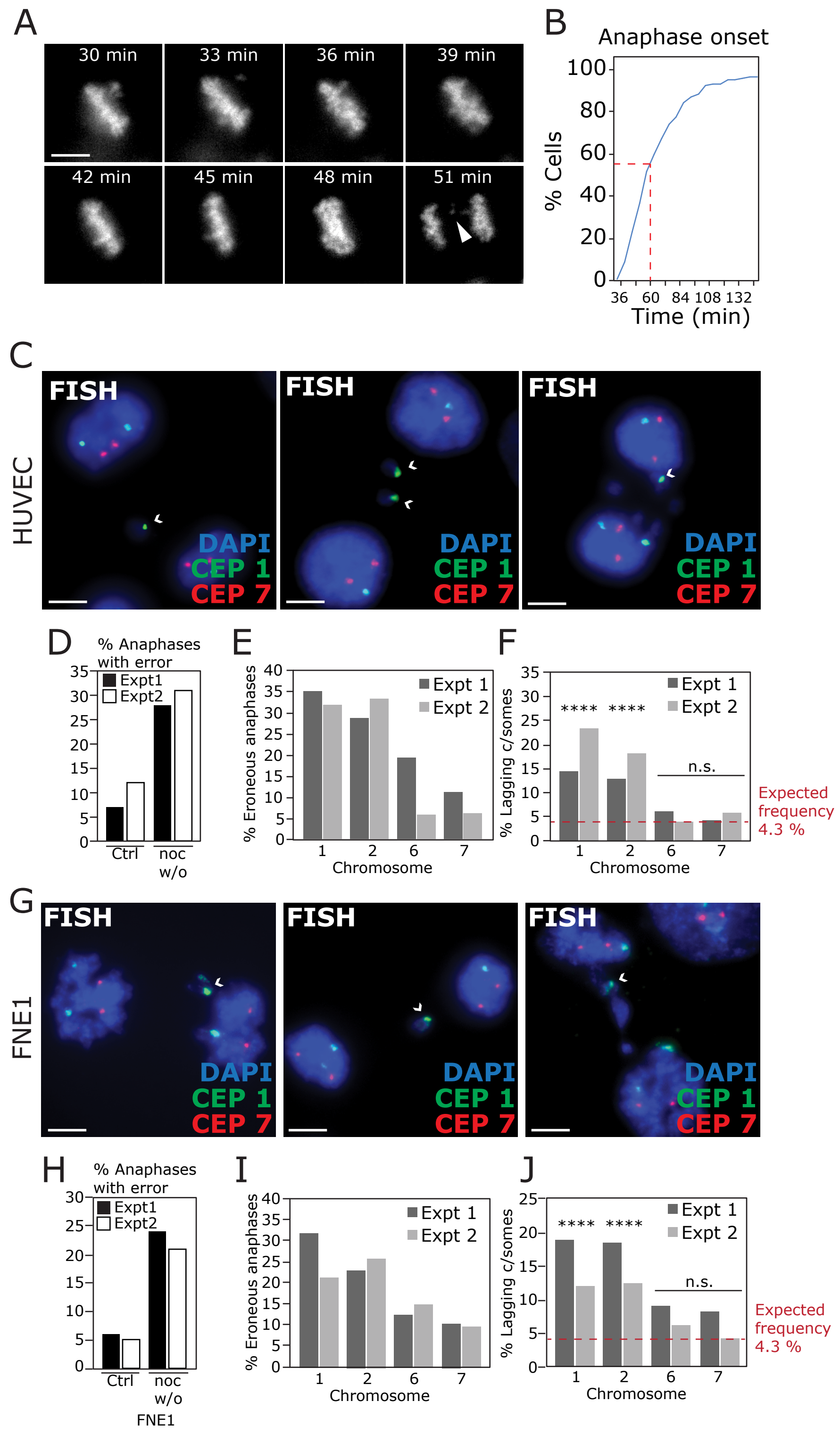

Figure S4 (relating to Figure 2). Chromosomes 1 and 2 are enriched in lagging chromosomes in multiple cell types. (A) Representative stills of movies of RPE1 cells stably expressing H2B-RFP, where filming began 30 minutes following washout from 8 hr nocodazole treatment. (**B**) Cumulative frequency plot of timing of anaphase onset following release from nocodazole is shown from 95 cells in total from two independent experiments. Red dashed line indicates percentage of cells in anaphase at 60 minutes. (**C**) FISH image of chromosome 1 (green) and chromosome 7 (red) from HUVEC cells treated with nocodazole then released for 1 hr. (**D**) % Anaphases with ≥1 lagging chromosome. (**E**) % Erroneous HUVEC anaphases (≥1 lagging chromosome) exhibiting lagging of chromosomes indicated. Results from 2 independent experiments are shown, 98 (CEP 1 and 7) and 52 (CEP 2 and 6) cells in total. (**F**) Quantification of % of lagging chromatids that are the chromosome indicated from erroneous anaphases (257 (CEP 1 and 2) and 147 (CEP 2 and 6) lagging chromosomes analysed in total. (**G**) FISH image of chromosome 1 (green) and chromosome 7 (red) from FNE1 (fallopian tube epithelial) cells treated with nocodazole then released for 1 hr. (**H**) % Anaphases with ≥1 lagging chromosome. (**I**) % Erroneous FNE1 anaphases (≥1 lagging chromosome) exhibiting lagging of chromosomes indicated. Results from 1 experiment are shown, 47-48 cells analysed per chromosome. (**J**) Quantification of % of lagging chromatids that are the chromosome indicated from erroneous anaphases. 85 (CEP 1 and 7) and 65 (CEP 2 and 6) lagging chromosomes analysed in total. P values in (F and J) were determined using a binomial test with Bonferroni multiple testing correction applied (significance considered for $p<0.0015$ (FNE1 and HUVEC)) (see Experimental procedures) $**<0.005$, $****<0.00005$. All scale bars 5 µm.

Supplemental Figure 5 (relating to Figure 3)

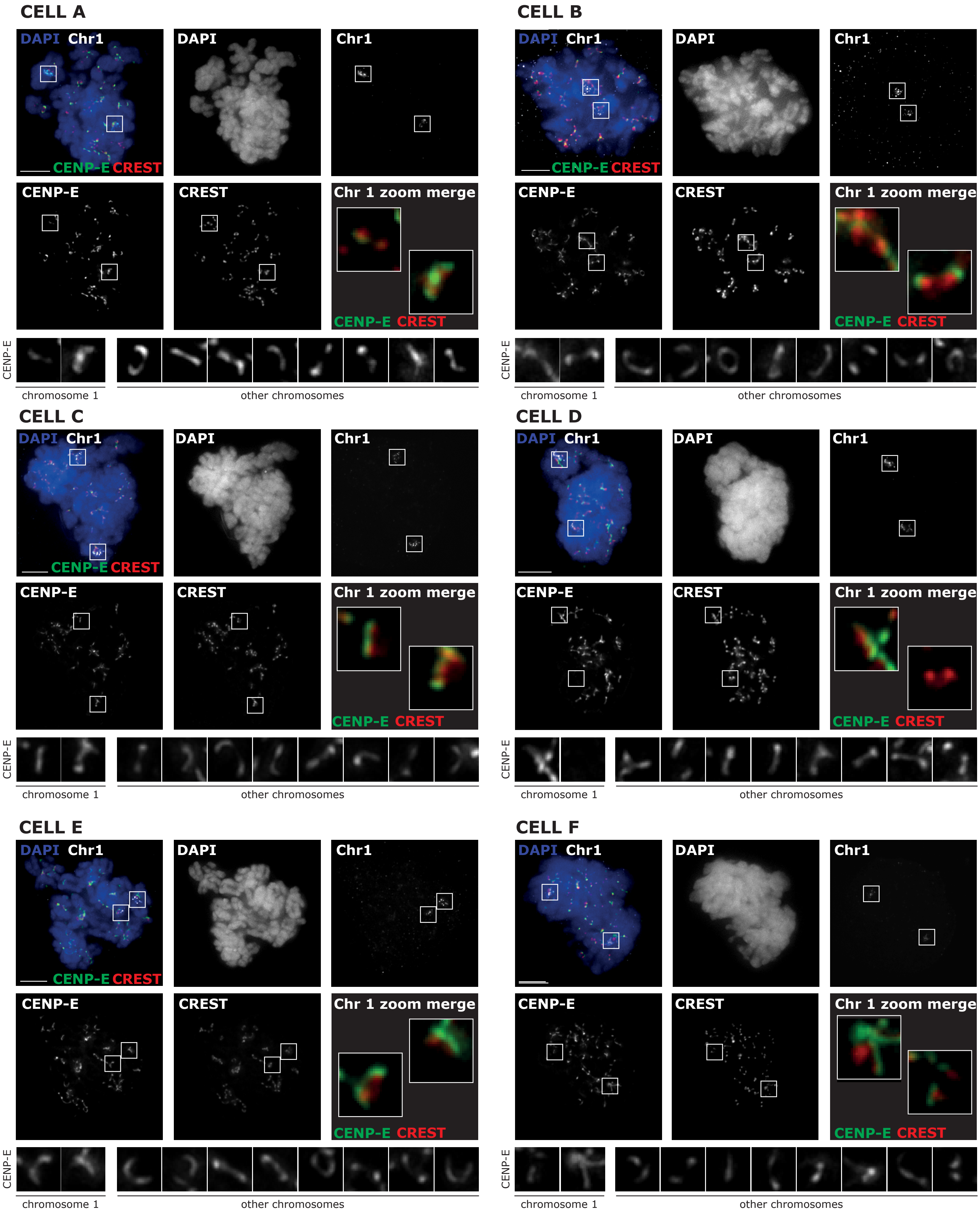

Figure S5 (relating to Figure 3). Chromosome 1 does not exhibit gross differences in kinetochore expansion following nocodazole treatment. Representative immunofluorescence-FISH images of cells treated with 8 hr nocodazole before fixation and staining with antibodies to CREST and CENP-E, and probing with CEP 1 to distinguish the centromere of chromosome 1 (white). Zooms indicate CENP-E and CREST signals at centromere pairs. All images are projections of 10 z-slices. Scale bars 5 μm.

Supplementary Figure 6 (relating to Figure 4)

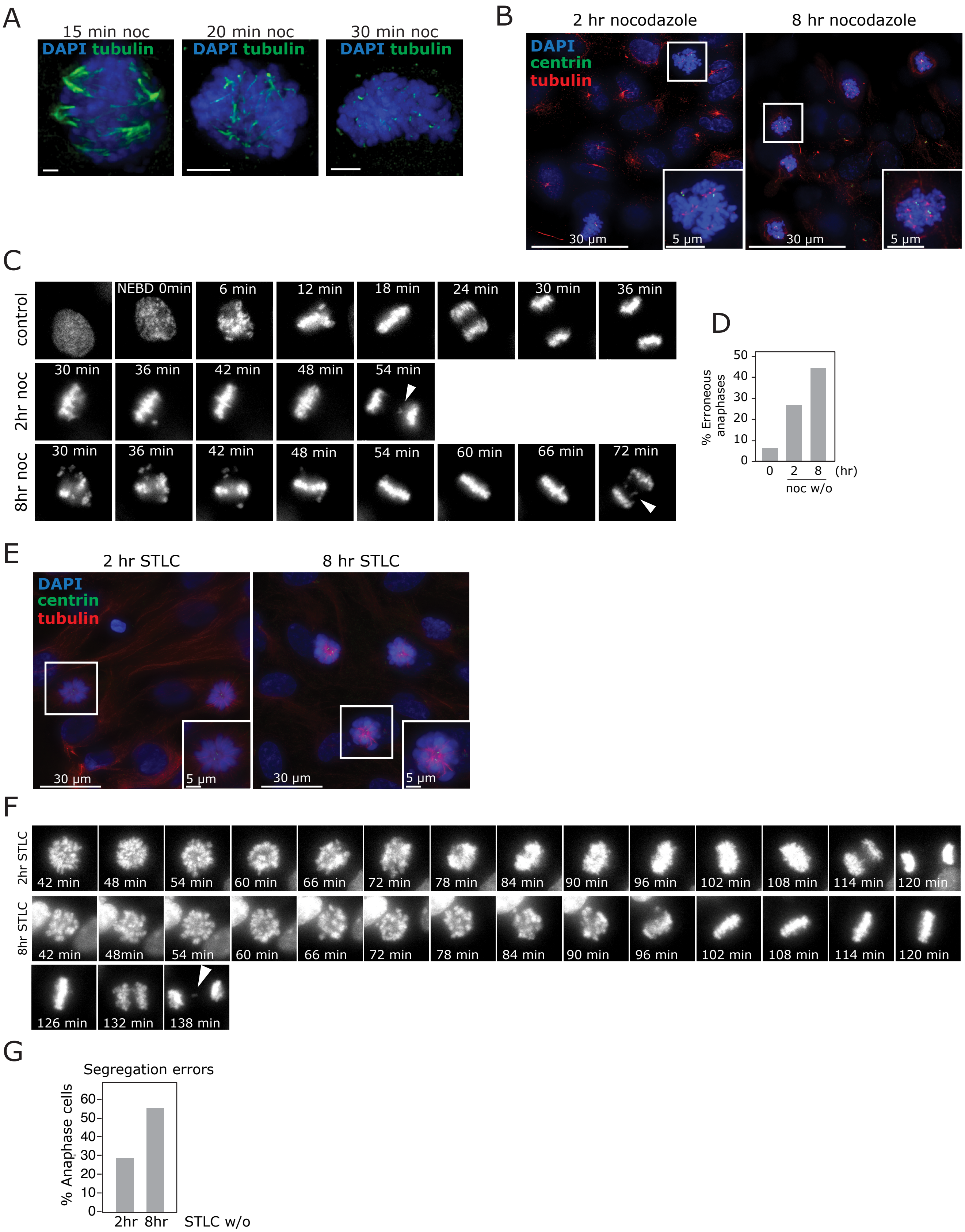

Figure S6 (relating to Figure 4). Chromosome segregation errors are promoted by increasing mitotic delay following nocodazole or Eg5 inhibition and release. (A) Representative immunofluorescence images of cells treated with nocodazole for times indicated before fixation and staining for antibodies to CREST and beta-tubulin. (**B**) Representative immunofluorescence images of cells treated with nocodazole for times indicated before fixation and staining for antibodies to beta-tubulin and centrin 3 (to mark centrosomes) to determine efficiency of MT depolymerisation. (**C**) Representative stills of movies of RPE1 cells stably expressing H2B-RFP, where filming began 30 minutes following washout from 2 or 8 hr nocodazole treatment (no washout). (**D**) Quantification of anaphases with lagging chromosomes from live cell movies (57, 57 and 95 cells cells in total from two independent experiments from 0, 2 and 8 hr nocodazole respectively). (**E**) RPE1 cells were fixed after 2 or 8 hr Eg5 inhibitor (STLC) (no washout) before staining with antibodies to beta-tubulin and centrin 3 to determine efficiency of MT depolymerisation. (**F**) RPE1 cells stably expressing H2B-RFP were filmed following release from 2 or 8 hr STLC treatment. Filming began 30 minutes after release. (**G**) Quantification of anaphases with lagging chromosomes from live cell movies (70 (2 hr STLC) and 85 (8 hr STLC) cells in total from two independent experiments). Images are whole cell projections. Scale bars are 5 µm unless otherwise indicated.

Supplemental Figure 7 (relating to Figure 5)

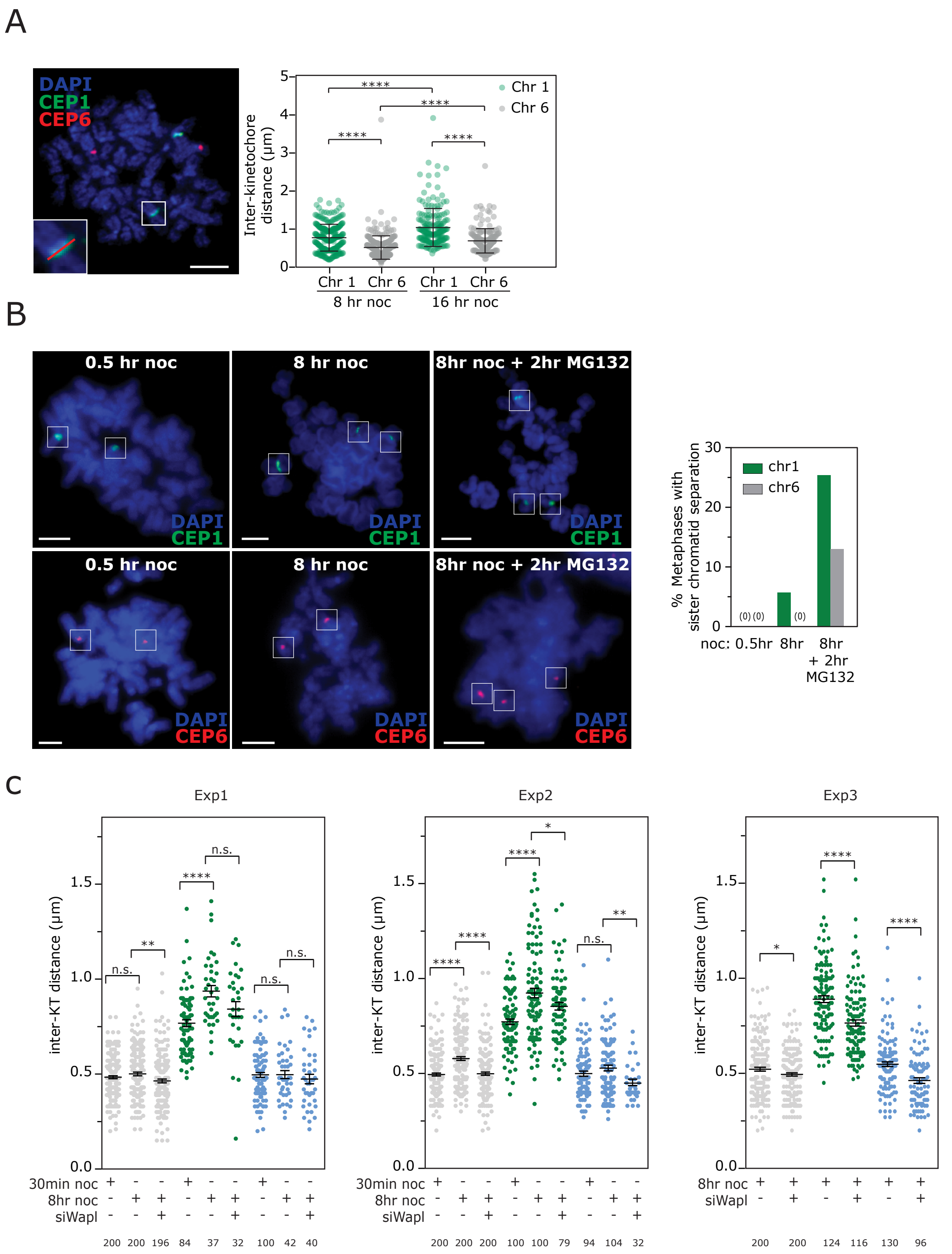

Figure S7 (relating to Figure 5). Chromosome 1 is prone to increased inter-centromere distance and sister chromatid separation. **(A)** RPE1 cells were treated with nocodazole for 8 or 16 hr then metaphase spreads were prepared by mitotic shake-off and pipetting onto glass slides (see Experimental procedures) before FISH with centromere enumeration probes as indicated. Inter-centromere distances were quantified from by measuring centromere pairs from edge-to-edge (see inset in (A)), (175-273 centromere pairs per condition from three (8 hr noc) or two (16 hr noc) independent experiments, mean and SD are shown). **(B)** Metaphase spreads were prepared from cells treated with nocodazole for times indicated by dropping onto glass slides (see Methods) before FISH centromere enumeration probes as indicated. A treatment of 8 hr nocodazole followed by 2 hr MG132 was included as a positive control. % Metaphases with completely separated sister chromatid pairs were quantified from at least 50 metaphases per chromosome per condition. **(C)** RPE1 cells were treated with non-targeting siRNA or siRNA targeted against Wapl for 39 hr before treatment with 8 hr nocodazole, (48 hr siRNA in total) then FISH using PNA (peptide nucleic acid) centromere-targeted probes (red) and specific centromere probes for chromosomes 1 and 2 (green). Note no PNA signal was visible at centromere 1 therefore these measurements were made using the centromere-specific probe signal. Three independent experiments are shown that comprise Figure 6c. Mean and SEM are shown. All statistical tests were unpaired t-tests (* $p<0.05$, ** $p<0.005$, **** $p<0.00005$).

Table S1 (relating to Figure 1). Chromosome characteristics.

Chromosome	Group	Centromere size (Mb)	Centromere coordinates	Total Genes On Chromosome	Gene density
1	A - metacentric chromosome	3.2	122,026,460-125,184,587	5078	20.31
2	A - metacentric chromosome	1.9	92,188,146-94,090,557	3862	15.96
3	A - metacentric chromosome	2.9	90,772,459-93,655,574	2971	15.01
4	B - sub-metacentric chromosome	2	49,708,101-51,743,951	2441	12.85
5	B - sub-metacentric chromosome	3.6	46,485,901-50,059,807	2578	14.16
6	C - medium-sized; meta-/submetacentric chromosome	1.3	58,553,889-59,829,934	3000	17.54
7	C - medium-sized; meta-/submetacentric chromosome	2.7	58,169,654-60,828,234	2774	17.45
8	C - medium-sized; meta-/submetacentric chromosome	1.8	44,033,745-45,877,265	2152	14.84
9	C - medium-sized; meta-/submetacentric chromosome	2.3	43,236,168-45,518,558	2262	16.39
10	C - medium-sized; meta-/submetacentric chromosome	1.9	39,686,683-41,593,521	2174	16.22
11	C - medium-sized; meta-/submetacentric chromosome	3.3	51,078,349-54,425,074	2920	21.63
12	C - medium-sized; meta-/submetacentric chromosome	2.4	34,769,408-37,185,252	2521	18.95
13	D - medium-sized; acrocentric chromosome (with rDNA)	2.1	16,000,001-18,051,248	1381	12.11
14	D - medium-sized; acrocentric	2.2	16,000,001-18,173,523	2055	19.21

	chromosome (with rDNA)				
15	D - medium-sized; acrocentric chromosome (with rDNA)	2.7	17,000,001-19,725,254	1814	17.78
16	E - small meta-/submetacentric chromosome	2	36,311,159-38,280,682	1920	21.33
17	E - small meta-/submetacentric chromosome	4.1	22,813,680-26,885,980	2432	29.30
18	E - small meta-/submetacentric chromosome	5.4	15,460,900-20,861,206	988	12.35
19	F - small metacentric chromosome	2.7	24,498,981-27,190,874	2481	42.05
20	F - small metacentric chromosome	3.6	26,436,233-30,038,348	1349	21.08
21	G - small acrocentric chromosome (with rDNA)	2.1	10,864,561-12,915,808	756	16.09
22	G - small acrocentric chromosome (with rDNA)	2.1	12,954,789-15,054,318	1172	22.98
X	C - medium-sized; meta-/submetacentric chromosome	3.8	58,605,580-62,412,542	2158	13.83
Y	G - small acrocentric chromosome	0.227	10,316,945-10,544,039	577	10.12

Table S1. Information regarding chromosome characteristics was deduced from NCBI Mapviewer database, annotation release 108. Centromere coordinates and size were gathered from the assembly regions specifically identified with the name "CEN".

Table S2 (relating to Figure 1). Centromere probes.

Chromosome	Probe	Chromosome Region	DNA Class	Notes
1	CEP1	1q12	Satellite III	Analysed by ImageStream (**Fig. 1g,h**)
2	CEP2	2p11.1-q11.1	α-satellite	Analysed by ImageStream (**Fig. 1g,h**)
3	CEP3	3p11.1-q11.1	α-satellite	Analysed by ImageStream (**Fig. 1g,h**)
4	CEP4	4p11.1-q11.1	α-satellite	Shows occasional cross-reactivity with a group C chromosome 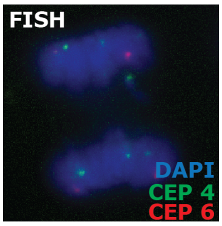 (ref:Cytocell). Therefore excluded from further use.
5	N/A			No specific centromeric probe available due to high sequence similarity between chromosomes 1, 5 and 19. Specific sub-telomere probe was tested but did not provide adequate signal to noise for ImageStream analysis.
6	CEP6	6p11.1-q11.1	α-satellite	Analysed by ImageStream (**Fig. 1g,h**)
7	CEP7	7p11.1-q11.1	α-satellite	Analysed by ImageStream (**Fig. 1g,h**)
8	CEP8	8p11.1-q11.1	α-satellite	Analysed by ImageStream (**Fig. 1g,h**)
9	CEP9	9q12	Satellite III	Analysed by ImageStream (**Fig. 1g,h**)
10	CEP10	10p11.1-q11.1	α-satellite	Analysed by ImageStream (**Fig. 1g,h**)
11	CEP11	11p11.1-q11.1	α-satellite	Analysed by ImageStream (**Fig. 1g,h**)
12	CEP12	12p11.1-q11.1	α-satellite	Analysed by ImageStream (**Fig. 1g,h**)
13	N/A			No specific centromeric probe available due to high sequence similarity between other acrocentric chromosomes. Specific sub-telomere probe was tested but did not provide adequate signal to noise for ImageStream analysis.
14	N/A			No specific centromeric probe available due to high sequence similarity between other acrocentric chromosomes. Specific sub-telomere probe was tested but did not provide adequate signal to noise for ImageStream analysis.

15	CEP15	15p11.1-q11.1	α-satellite	Analysed by ImageStream (**Fig. 1g,h**)
16	CEP16	16p11.1-q11.1	α-satellite	Analysed by ImageStream (**Fig. 1g,h**)
17	CEP17	17p11.1-q11.1	α-satellite	Analysed by ImageStream (**Fig. 1g,h**)
18	CEP18	18p11.1-q11.1	α-satellite	Analysed by ImageStream (**Fig. 1g,h**)
19	N/A			No specific centromeric probe available due to high sequence similarity between chromosomes 1, 5 and 19. Specific sub-telomere probe was tested but did not provide adequate signal to noise for ImageStream analysis.
20	CEP20	20p11.1-q11.1	α-satellite	Unequal intensity between homologous alleles sometimes observed. Analysed by ImageStream (**Fig. 1g,h**)
21	N/A			No specific centromeric probe available due to high sequence similarity between other acrocentric chromosomes. Specific sub-telomere probe was tested but did not provide adequate signal to noise for ImageStream analysis.
22	N/A			No specific centromeric probe available due to high sequence similarity between other acrocentric chromosomes. Specific sub-telomere probe was tested but did not provide adequate signal to noise for ImageStream analysis.
X	CEPX	Xp11.1-q11.1	α-satellite	Analysed by ImageStream (**Fig. 1g,h**)
Y	N/A			

All CEP probes were from Cytocell.

Supplemental experimental procedures.

Cell lines

hTERT-RPE-1 cells were cultured in DMEM Nutrient Mixture F12 Ham (Sigma); BJ cells in DMEM high glucose (Sigma). Media for both was supplemented with 10% FBS and 100 U Penicillin/Streptomycin. RPE1 and BJ cells were subjected to STR profiling to verify their identity using the cell line authentication service from Public Health England. HUVEC cells were cultured in Huvec media (Medium 199, Gibco; 20% FBS; Endothelial Cell Growth Supplement, Sigma; 10U/ml Heparin, Sigma). FNE1 (University of Miami) cells were grown in FOMI media (University of Miami) supplemented with cholera toxin (Sigma).

ImageStream cytometry analysis

All samples were analysed on the ImageStream cytometer by excitation with the blue laser with a power of 100 mW at a 'high' flow speed. Data obtained by the ImageStream were analysed in IDEAS 6.2 (Merck Millipore). Samples for each chromosome and experimental condition were obtained separately and contained within a single data file. For each sample a minimum of 500, and a maximum of 40,000, cells were analysed. Raw data files were opened in the IDEAS software package and the built-in compensation matrix applied. This correction is necessary to remove fluorescent noise introduced from the spatial alignment between channels, the flow speed, camera background normalisation and the level of brightfield gain. During acquisition, the EDF element was used to increase the focus range from 4 µm to 16 µm, allowing close to 100% of cells to be focused. Single cells are distinguished from cell aggregates by low area and high aspect ratio. The gating of single cells was manually verified by visual observation of brightfield images in the selected region. Plotting the Gradient root mean squared (RMS) value of the brightfield channel allowed only cells that were in-focus to be analysed. In-focus cells have a high Gradient RMS value. For some samples, where the hybridisation efficiency was less, a further gate was applied to select for only cells in the sample above a threshold of probe signal intensity. This was achieved by plotting the total intensity of fluorescence in each cell, versus the Raw Max Pixel intensity within the cell. Cells with hybridised probe have an average total fluorescence, and a high Raw Max Pixel intensity. Single, in-focus, hybridised cells were then analysed for the chromosomal content of a particular chromosome by applying a 'spot mask' and 'spot counting' feature to the centromere probe signals for each image. The masking parameters were determined on user-defined variables: the radius of the spot and the spot-to-background ratio (STBR). The STBR is the spot pixel value divided by the background fluorescence of the bright detail image. The spot mask therefore denotes a region that is of appropriate area to be considered a centromeric signal, and the boundary at which the signal diminishes. Where the radius value is x, this suggests that the denoted area of a single spot should have a minimum value of 2x+1 pixels. Regions that satisfy the spot mask criteria in single cells are enumerated by the spot-counting wizard. For the wizard to accurately determine chromosome ploidy, truth populations were denoted for both 2n-1 and 2n+1 cells for a minimum of 25 images. The wizard then compiles the common features for over 100 elements and assigns each image a spot count.

The images obtained of CEP spots are 2D projections of 3D images, to encompass the entire volume of the nucleus. If a cell is aligned so that the two centromere signals are in the same plane, they sometimes appear as a single focus, because they overlap following image projection. To correct for this, CEP signal intensity was plotted as a histogram from the original spot count data which correlates with the amount of probe hybridised, rather than the spot count. Disomic cells had a medium (M) intensity of hybridisation signal intensity, representing two spots. Cells with one spot that had lost a chromosome will fall below the value represented by two standard deviations above the mean fluorescent intensity; cells that had gained a chromosome will fall above two standard deviations of the mean of the hybridisation signal intensity. Events that are classified as one spot by the software usually fell into the medium range for intensity in the majority of cases. This suggests that, for the reasons stated above, they are disomic cells with aberrant ploidy-spot relationship. Cells designated as one spot that fell outside the 2 standard deviation window were deemed to be true monosomies. Cells designated as 2n+1 by the spot-counting wizard were manually verified by visual inspection of each image and correlating it with the 2 standard deviation cut-off above the mean diploid fluorescence intensity. To verify this gating strategy RPE1 (diploid) and HCT116 (monosomic for the X chromosome) cells were probed with combinations of probes to yield populations with one (HCT116 X centromere probe), two (RPE1 X centromere probe), or three (HCT118 X plus 18 centromere probes) centromere signals per cell, before plotting of intensity histograms (**Figure S2**).

Single cell Sequencing

Single nuclei were isolated and stained with 10 µg/mL propidium iodide and 10 µg/mL Hoechst. Single nuclei with low Hoechst/PI fluorescence (G1 population) were sorted into 96-well plates containing freezing buffer using a FACSJazz (BD Biosciences). Pre-amplification-free single-cell whole genome sequencing libraries were prepared using a Bravo Automated Liquid Handling Platform (Agilent Technologies, Santa Clara, CA, USA), followed by size-selection and extraction from a 2% E-gel EX (Invitrogen). Single-end 84 nt sequence reads were generated using the NextSeq 500 system (Illumina, San Diego, CA, USA) at 384 single-cell DNA libraries per

flow cell. Demultiplexing based on library-specific barcodes and conversion to fastq format was done using bcl2fastq (v1.8.4, Illumina). Duplicate reads were called using BamUtil (v1.0.3). Demultiplexed reads were aligned to the GRCh38 reference genome using bowtie (v2.2.4) and only uniquely mapped reads (MAPQ>10) were used for further analysis. Copy number annotation was performed using AneuFinder (v1.4.0). Sequence reads are determined as non-overlapping bins with an average length of 1 Mb, a GC correction is applied, and binned sequences are analysed using a Hidden Markov model to determine the most likely copy number states. To negate the inherent sample variation introduced by sequencing single cells, a stringent quality control step was included that uses multivariate clustering to exclude libraries of insufficient quality. Chromosome copy number is plotted as a genome-wide state with clustering of cells based on the similarity of copy number profiles.

<u>Statistical analysis</u>

Asterisks have been used to denote the significance value between experimental conditions adhering to the following nomenclature: p<0.05 (*); p<0.005 (**); p<0.0005 (***); p<0.00005 (****). To test whether specific chromosomes displayed mis-segregation more often than others in the ImageStream analysis we performed a Chi-squared test using MATLAB (R2016B). The expected number of mis-segregations was set to the average of the observed rates of mis-segregation multiplied by the number of cells. This estimate was used because we do not observe the total number of mis-segregations M (only 1 or 2 chromosomes are labelled per cell) and therefore cannot simply use $M/23$. For significant tests, we used post hoc Binomial tests, with Bonferroni multiple testing correction, to identify deviating chromosomes with the same rate as in the Chi-squared. For the lagging experiments, we used the same statistical approach but with expected number of lagging chromosomes $M/23$ since we observed all lagging chromosomes in every cell.

Supplemental references.

Di Nicolantonio, F., Arena, S., Gallicchio, M., Zecchin, D., Martini, M., Flonta, S.E., Stella, G.M., Lamba, S., Cancelliere, C., Russo, M., *et al.* (2008). Replacement of normal with mutant alleles in the genome of normal human cells unveils mutation-specific drug responses. Proceedings of the National Academy of Sciences of the United States of America *105*, 20864-20869.

Zhang, C.Z., Spektor, A., Cornils, H., Francis, J.M., Jackson, E.K., Liu, S., Meyerson, M., and Pellman, D. (2015). Chromothripsis from DNA damage in micronuclei. Nature *522*, 179-184.
